# A guide to sunflowers: floral resource nutrition for bee health and key pollination syndromes

**DOI:** 10.3389/fpls.2025.1552335

**Published:** 2025-05-01

**Authors:** Salena Husband, Katarina Cankar, Olivier Catrice, Stan Chabert, Silvio Erler

**Affiliations:** ^1^ Institute for Bee Protection, Julius Kühn-Institute (JKI) – Federal Research Centre for Cultivated Plants, Braunschweig, Germany; ^2^ Zoological Institute, Technische Universität Braunschweig, Braunschweig, Germany; ^3^ Wageningen Plant Research, Business Unit Bioscience, Wageningen University and Research, Wageningen, Netherlands; ^4^ Université de Toulouse, Institut National de Recherche pour l’Agriculture, l’Alimentation et l’Environnement (INRAE) Unité Mixte de Recherche (UMR) Le Laboratoire des Interactions Plantes-Microbes-Environnement (LIPME), Castanet-Tolosan, France

**Keywords:** floral rewards, *Helianthus annuus*, nectar, plant-pollinator interactions, pollen, pollination syndromes, pollinator health, pollinator nutrition

## Abstract

Sunflower, *Helianthus annuus* L., is a prominent global oilseed crop with rising cultivation and appeal as a bee-friendly plant by providing abundant floral resources for pollinators. Mass-flowering crops can increase the availability of resources, and sunflower is a good opportunity to relieve pollen scarcity during the late summer in agricultural landscapes. Yet this should be taken with caution as they also provide a homogeneous source of nutrition. This study aimed to review and summarize the nutritional profile of sunflower pollen, nectar, bee bread, and honey, while assessing their effects on bee survival, development, and health. Furthermore, we present here the general state of knowledge on additional pollinator syndromes that extend beyond floral resources, including those influencing pollinator visual and olfactory attraction. We found that while sunflower pollen’s nutritional quality is questioned due to lower protein and amino acid deficiencies, its nutrient content, like nectar sugars, had large variability. Sunflower pollen consumption showed mixed effects on *Apis mellifera* and *Bombus* species, sometimes negatively impacting development and survival. However, studies have conveyed a positive impact on bee health as sunflower pollen consistently reduced the infection intensity of the gut parasite, *Crithidia bombi*, in *Bombus* species. This probes the question on defining the quality of floral resources, emphasizing the need for caution when categorizing sunflower as a low quality nutritional resource. This review also outlines the importance of sunflower nectar characteristics (sugar content and volume) and floral morphology (flower pigmentation and corolla length) on pollinator foraging preferences. A prominent knowledge gap persists regarding nectar chemistry and sunflowers’ extensive volatile profile to better understand the pollination syndromes that drive its pollinator interactions.

## 1 Introduction

Floral resource availability and nutritional composition play an essential role in pollinators’ development and survival. Our understanding of bee nutrition and foraging behaviors encompasses mainly honey bees, *Apis mellifera* (Haydak, 1970; Tsuruda et al., 2021; Winston, 1987) and more recently bumble bees, *Bombus* spp (Goulson, 2003; Prŷs-Jones and Corbet, 2011). Less is known regarding the species-specific balance between macro- and micronutrients and in regards to wild bee species. The honey bees’ diet consists mainly of pollen and nectar, including their stored products in the hive known as bee bread and honey. Pollen and bee bread are the primary sources of nutrition in the form of proteins, lipids, vitamins, and minerals that are crucial for normal growth and development, reproduction, brood rearing, and health (Winston, 1987). Bee bread differs from freshly collected pollen as it is stored in the hive with the addition of nectar or honey; various salivary enzymes; and diverse bacterial species, some responsible for natural fermentation (Ghosh et al., 2022; Winston, 1987). Nectar fulfils the essential energetic resource requirements, primarily through glucose, fructose, and sucrose needed for foraging and flight (Winston, 1987). Honey, stored nectar, is similarly consumed for its carbohydrates. Honey is characterized by its high sugar concentration (~80%); diverse sugar spectrum dependent on the nectar’s floral origin; bee-secreted enzymes; and antibacterial properties (Crane, 1975; Molan, 1992). All bee collected and processed products might be applied and shared within the hive for adult bee and larvae medication (self-medication, allo-medication), which means they are consumed for disease prevention or therapy, to cure infected individuals or boosting the immune system (Erler and Moritz, 2016; Erler et al., 2024). This hypothesis has been proven experimentally for several cases using honey bees and bumble bees of different species (see sections 3.1 and 3.3 of this manuscript).

The nutritional breakdown of pollen and nectar, and subsequently bee bread and honey, varies depending on the plant species (Pamminger et al., 2019a, b; Stephen et al., 2024). It is under current debate how to determine a nutritionally balanced diet for different pollinators (Bryś and Strachecka, 2024; Vaudo et al., 2024), yet this remains difficult when their specific nutritional requirements are still uncertain. The traditional concept, basing the quality of a pollinator’s diet solely on the crude protein and essential amino acid content, has been scrutinized for general validity. Rather, it might be essential to consider other factors such as protein:lipid ratios which may impact foraging decisions (Vaudo et al., 2020) and stoichiometric ratios of mineral nutrients (Filipiak et al., 2017). A rising topic in bee health research is the role of plant secondary metabolites ingested by bees from pollen, nectar, honey, and bee bread, including alkaloids, polyphenols, terpenoids, and many others, but our knowledge is limited to honey bees and bumble bees (Barberis et al., 2023). Secondary metabolites have shown to boost cognitive functioning (Baracchi et al., 2017) and immunity (Palmer-Young et al., 2017); or even restrict development (Rivest et al., 2024). Secondary metabolites can shape insect immunity against parasites and pathogens (Erler et al., 2024; Fitch et al., 2022). These metabolites can have direct effects on the host's immune system or exert antimicrobial effects independently, thereby indirectly enhancing insect fitness. We are just at the beginning of understanding how these metabolites may interact with pollinators.

It is acknowledged that mono-diets, consisting of a single pollen source, are not considered well-balanced and social bees need a mix of plant sources to balance the nutrients that are missing in some sources, but present in others, to maintain healthy colonies (Alaux et al., 2010; Di Pasquale et al., 2013, 2016). A mixed pollen diet is presumable also important for generalist solitary species, however research is lacking. Modern agricultural practices, monoculture, and pollen scarcity, especially in late summer, may be a key factor in the combined stressors impacting seasonal survival and contributing to pollinator decline (Balvino-Olvera et al., 2024; Di Pasquale et al., 2016; Dolezal et al., 2019; Donkersley et al., 2014; Harris et al., 2024; Lau et al., 2023; Requier et al., 2015, 2017; Wright et al., 2024). Mass-flowering crops can increase the availability of resources for flower-visiting insects in agricultural environments (Di Pasquale et al., 2016; Harris et al., 2024; Holzschuh et al., 2013; Westphal et al., 2003), especially sunflower (*Helianthus annuus* L.) which blooms during the critical summer period (Requier et al., 2015, 2017). The impact of mass-flowering crops should be put into perspective since they only offer a homogeneous resource over a limited period (Di Pasquale et al., 2013, 2016; Goulson et al., 2015; Potts et al., 2010, 2016). This makes it important to understand in greater detail the nutritional needs of pollinators, the nutritional value that large-scale flowering crops can provide, and how it may impact the development and health of pollinators.

**Figure 1:**
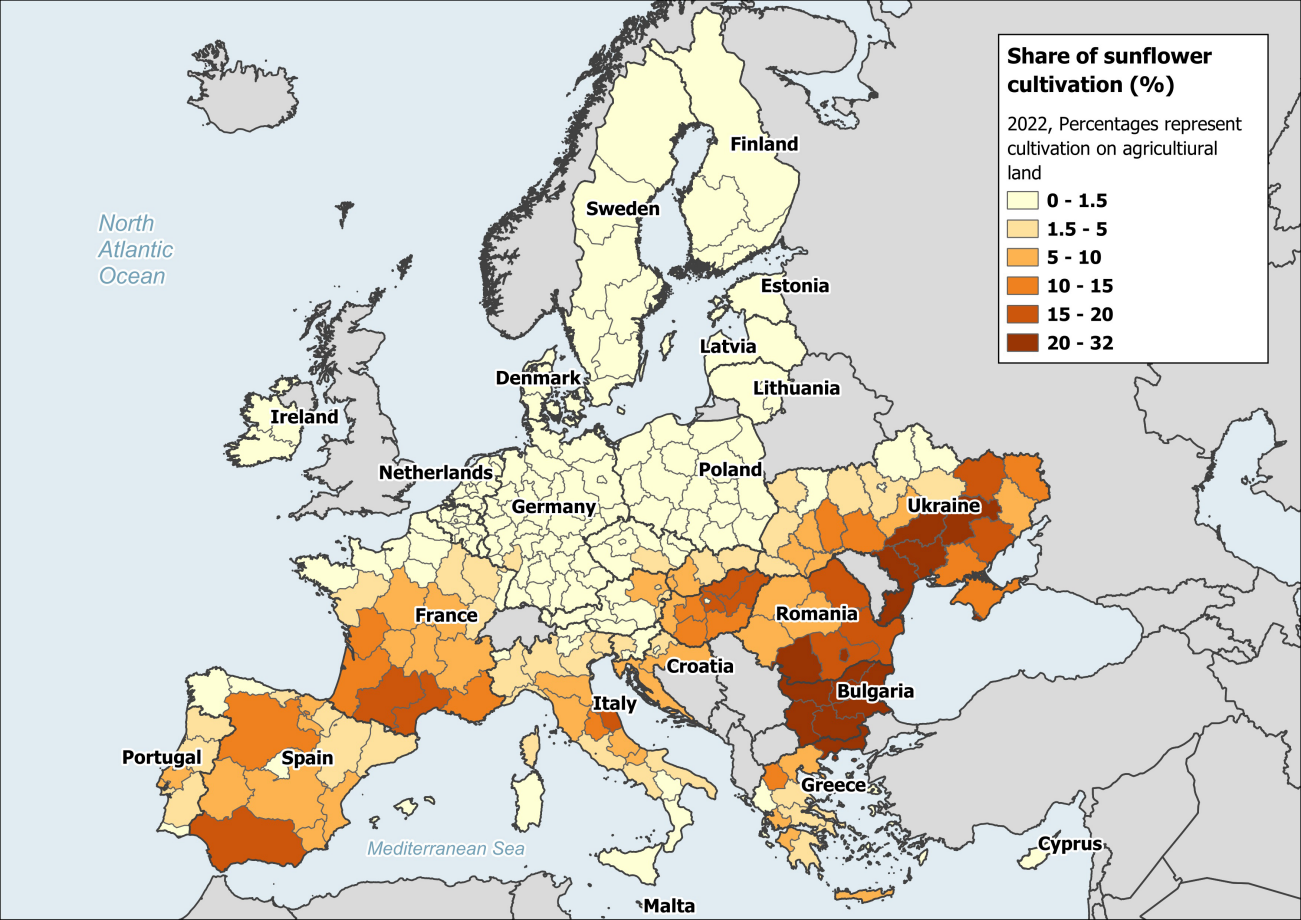
Share of sunflower cultivation on agricultural land in 2022. Source: Data extracted from European Commission, Joint Research Centre (JRC) (2022), for sunflower cultivation in EU 27 member states and Ukraine. (doi: 10.2905/555e5d1d-1aae-4320-a716-2e6d18aa1e7c).

Sunflower is one such late-flowering, large scale crop surpassing 29 Mha and seed production reaching 54 million tons in 2022 (FAO, 2024). Across Europe, the share of sunflower production in agricultural landscapes is primarily in Eastern and Southern regions, such as Ukraine, Romania, Bulgaria, France, and Spain (**Figure 1**). With the increasing demand of sunflower production and climate change, there is an expected shift in sunflower production towards Northern European regions. Sunflower production relies partly or completely on insect mediated pollen transfer for oilseed, confection and hybrid seed production (Chabert et al., 2022; Mallinger et al., 2019; Mallinger and Prasifka, 2017a; Oz et al., 2009). It is known to attract a diversity of wild and managed bees (references reviewed in Brown and Cunningham, 2019; Chabert et al., 2022), with higher diversity observed in North America, which is the native range of the *Helianthus* species (Heiser et al., 1969; Kantar et al., 2015; Mandel et al., 2011; Seiler and Jan, 2010).

Pollination syndromes is a general term used to describe floral traits mediating interaction between plants and pollinators (Knudsen and Tollsten, 1993). Pollen and nectar resources are called ‘rewards’ or ‘primary attractants’ (Armbruster, 2012; Shivanna, 2014) and as such were shown, with nectar access, to influence foraging preference in sunflowers; including preferences for different cultivars (Bergonzoli et al., 2022; Cerrutti and Pontet, 2016; Freund and Furgala, 1982; Mallinger and Prasifka, 2017b; Tepedino and Parker, 1982), nectar quantity (Mallinger and Prasifka, 2017b), nectar sucrose content (Pham-Delègue et al., 1990, 1994; but see Mallinger and Prasifka, 2017b), pollen production (Mallinger and Prasifka, 2017b), and nectar access through corolla length (Ferguson et al., 2021; Mallinger and Prasifka, 2017b; Portlas et al., 2018). Other flower traits are defined as ‘secondary attractants’, in the sense that they are cues associated with floral resources innately or through learning by bees (Armbruster, 2012; Frachon et al., 2021; Knauer and Schiestl, 2015; Ortiz et al., 2021; Shivanna, 2014; Wright and Schiestl, 2009). Such floral cues were associated with bee foraging preferences in sunflower, including floral volatile organic compounds (VOCs) (Pham-Delègue et al., 1986, 1990) and floral pigmentation (Todesco et al., 2022). This makes sunflower a particularly interesting crop which has gained renewed attention to enrich ecosystems by providing plentiful floral resources for bees in agricultural dominant landscapes. With the importance of sunflower in agricultural production and the substantial increase in cultivation, it is important to grasp the potential nutritional value of sunflower floral resources for pollinator communities and the pollinator syndromes which impact sunflower-pollinator interactions.

The aim of this review is to provide a foundation for sunflower nutritional research for pollinators and summarize the current state of knowledge on the chemistry of sunflower floral resources (pollen and nectar) and processed hive products (honey and bee bread). We also provide a comprehensive overview on targeted sunflower research investigating floral resource consumption on bee survival, development, and health. Furthermore, our review encompasses pollinator syndromes beyond floral rewards, examining additional factors that may shape pollinator attraction and foraging preferences in sunflowers. By doing so, this review seeks to identify future research directions and knowledge gaps to improve our understanding of sunflower plant-pollinator interactions.

## 2 Chemistry of sunflower floral resources, bee bread, and honey

### 2.1 Pollen

Sunflowers are often considered a “low-quality” resource since the crude protein content falls on the lower scale (Nicolson and Human, 2013; Pamminger et al., 2019a). Bee collected sunflower pollen protein content shows large variance ranging from 7.32–19.4 g/100 g, excluding the outlier 0.49 g/100 g from Stephen et al. (2024). Interestingly, hand-collected pollen had a higher range (11.6–26.5 g/100 g) (**Table 1**). In general, Asteraceae species crude protein has been reported in lower ranges (Vaudo et al., 2024), between 12–14% (Roulston et al., 2000) and 5.2–23.7% (Vaudo et al., 2020). In any case, valuing quality based on protein content alone should be taken with caution as wide ranges for crude protein values exists even for commonly reported “high-quality” pollen sources. For example, the crude protein from *Brassica* spp. pollen was reported between 22.2–33.8% by Pamminger et al. (2019a) but only 13.6% by Vaudo et al. (2020).

**Table 1:** Literature values of compositional parameters reported for *Helianthus annuus* bee- and hand-collected pollen and bee bread.

*H. annuus*	Pollen	Bee bread	References	
			Pollen	Bee bread
Ash (g/100 g DM)	BC: 1.61–2.01 HC: 3.45/5.59	1.54	BC: Kostić et al., 2020; Nicolson and Human, 2013 ^**^; Taha, 2015; Yang et al., 2013	Nicolson and Human, 2013
Carbohydrates (g/100 g DM)	BC: 45.2–82.0 HC: 17.7–62.6	80.2	BC: Conti et al., 2016; Kostić et al., 2020; Nicolson and Human, 2013 ^**^ HC: Russo et al., 2019; Treanore et al., 2019	Nicolson and Human, 2013
Crude protein (g/100 g DM)	BC: (0.49^*^) 7.32–19.4 HC: 11.6–26.5	13.3	BC: Conti et al., 2016; Kostić et al., 2020; Nicolson and Human, 2013 ^**^; Pernal and Currie, 2000; Stephen et al., 2024; Taha, 2015; Taha et al., 2019; Tasei and Aupinel, 2008; Vaudo et al., 2020 ^**^; Yang et al., 2013; Zhou et al., 2024 HC: Russo et al., 2019; Treanore et al., 2019	Nicolson and Human, 2013
Lipids (g/100 g DM)	BC: (0.34^*^) 1.52–8.26 HC: 7.46–12.5	4.98	BC: Nicolson and Human, 2013 ^**^; Stephen et al., 2024; Taha, 2015; Vaudo et al., 2020 ^**^; Zhou et al., 2024 HC: Russo et al., 2019; Shakya and Bhatla, 2010; Treanore et al., 2019	Nicolson and Human, 2013
Moisture content (g/100 g WM)	BC: 17.7–25.0 HC: 6.78/8.36	16.1	BC: Kostić et al., 2020; Nicolson and Human, 2013 ^**^; Pernal and Currie, 2000	Nicolson and Human, 2013

When necessary, data was extracted directly from plots using plotdigitizer.com. BC, bee-collected pollen; DM, dry mass; HC, hand-collected pollen; WM, wet mass. ^*^ Indicates an outlier; ^**^ Indicates the reference investigated both bee- and hand-collected pollen.

Amino acid profiles are important to consider in terms of protein quality. Based on the essential amino acid requirements for honey bees detailed in De Groot (1953), sunflower pollen was reported to have below required amounts of isoleucine, methionine, and tryptophan; and occasional deficiencies in arginine and phenylalanine (**Table 2**). It is not uncommon for pollen to have deficiencies in one or more amino acids, as it is the case for plant species within the Fabaceae and Boraginaceae families (Jeannerod et al., 2022). This review summarized only protein-bound amino acids to limit variability, since free amino acids may more readily be influenced or lost by different methods of pollen handling. Therefore, the amino acid content is not the absolute content available to bees, as other studies have shown that free amino acids contribute an additional 3.49–6.06% depending on species (McAulay and Forrest, 2019). It is also known that amino acids vary for the same species depending on analysis method (Jeannerod et al., 2022). Abiotic conditions, such as fertilization levels, were also found to be positively correlated with total amino acid content in pollen (Leach et al., 2021). Therefore, values should be interpreted with caution as the deficient amino acids may not be realistic in all natural settings, especially when free amino acid availability and other environmental factors may impact composition.

**Table 2:** Literature values for amino acids reported in *Helianthus annuus* bee- and hand-collected pollen, bee bread, and honey.

*H. annuus*: Amino acids (g/100 g protein)	Pollen	Bee bread	Honey	References		
				Pollen	Bee bread	Honey
Arginine (3.0)^1^	BC: 2.56/4.18; 10.5^*^/16.7^*^ HC: 4.35/4.38	4.77	0.041; 0.014^*^/0.057^*^	BC: McAulay and Forrest, 2019; Nicolson and Human, 2013 ^**^; Taha et al., 2019; Yang et al., 2013	Nicolson and Human, 2013	Cotte et al., 2004b; Kečkeš et al., 2013; Mohammed and Babiker, 2010
Histidine (1.5)	BC: 4.00/5.66; 6.9^*^/7.74^*^ HC: 5.44/5.52	5.75	0.007; 0.011^*^/0.016^*^	BC: McAulay and Forrest, 2019; Nicolson and Human, 2013 ^**^; Taha et al., 2019; Yang et al., 2013	Nicolson and Human, 2013	Cotte et al., 2004b; Kečkeš et al., 2013; Mohammed and Babiker, 2010
Isoleucine (4.0)	BC: 3.04–3.88; 5.1^*^/14.5^*^ HC: 3.93/3.95	4.01	0.044; 0.009^*^	BC: McAulay and Forrest, 2019; Nicolson and Human, 2013 ^**^; Taha et al., 2019; Yang et al., 2013	Nicolson and Human, 2013	Cotte et al., 2004b; Mohammed and Babiker, 2010
Leucine (4.5)	BC: 6.32/6.35; 7.9^*^/21.7^*^ HC: 6.35/6.55	6.55	0.051; 0.005/0.031^*^	BC: McAulay and Forrest, 2019; Nicolson and Human, 2013 ^**^; Taha et al., 2019; Yang et al., 2013	Nicolson and Human, 2013	Cotte et al., 2004b; Kečkeš et al., 2013; Mohammed and Babiker, 2010
Lysine (3.0)	BC: 4.56–6.29; 7.0^*^/17.9^*^ HC: 6.38/6.85	5.98	0.046; 0.007/0.011^*^	BC: McAulay and Forrest, 2019; Nicolson and Human, 2013 ^**^; Taha et al., 2019; Yang et al., 2013	Nicolson and Human, 2013	Cotte et al., 2004b; Kečkeš et al., 2013; Mohammed and Babiker, 2010
Methionine (1.5)	BC: 0.24/0.31; 1.2^*^/7.61^*^ HC: 0.54	0.27	0.039; 0.004^*^	BC: McAulay and Forrest, 2019; Nicolson and Human, 2013 ^**^; Taha et al., 2019; Yang et al., 2013	Nicolson and Human, 2013	Cotte et al., 2004b; Mohammed and Babiker, 2010
Phenylalanine (2.5)	BC: 0.76/3.90; 4.6^*^/15.1^*^ HC: 3.90/3.91	3.94	0.044; 0.021/0.093^*^	BC: McAulay and Forrest, 2019; Nicolson and Human, 2013 ^**^; Taha et al., 2019; Yang et al., 2013	Nicolson and Human, 2013	Cotte et al., 2004b; Kečkeš et al., 2013; Mohammed and Babiker, 2010
Threonine (3.0)	BC: 2.80–4.38; 4.3^*^/13.2^*^ HC: 4.29/4.30	4.51	0.040; 0.011/0.038^*^	BC: McAulay and Forrest, 2019; Nicolson and Human, 2013 ^**^; Taha et al., 2019; Yang et al., 2013	Nicolson and Human, 2013	Cotte et al., 2004b; Kečkeš et al., 2013; Mohammed and Babiker, 2010
Tryptophan (1.0)	BC: 0.17–1.83; 3.61^*^/7.0^*^ HC: 0.26	0.14	0.002^*^	BC: McAulay and Forrest, 2019; Nicolson and Human, 2013 ^**^; Taha et al., 2019; Yang et al., 2013	Nicolson and Human, 2013	Cotte et al., 2004b
Valine (4.0)	BC: 4.17–5.46; 6.4^*^/17.8^*^ HC: 4.33/4.63	4.44	0.049; 0.006/0.013^*^	BC: McAulay and Forrest, 2019; Nicolson and Human, 2013 ^**^; Taha et al., 2019; Yang et al., 2013	Nicolson and Human, 2013	Cotte et al., 2004b; Kečkeš et al., 2013; Mohammed and Babiker, 2010

When necessary, data was extracted directly from plots using plotdigitizer.com. BC, bee-collected pollen; HC, hand-collected pollen. ^1^Number in parenthesis represent the minimal requirements for honey bees based on De Groot (1953); ^*^ Units (µg/mg) ^**^ Indicates the reference investigated both bee- and hand-collected pollen.

Pollen lipid content is another important parameter to consider for bee nutrition. Not all lipids are synthesized endogenously (Arien et al., 2018; Winston, 1987), therefore it is vital to investigate their detailed composition obtained in bees’ diets. The lipid content of bee-collected sunflower pollen (**Table 1**), is reported within the range 1.52–8.26%, excluding the outlier 0.34% from Stephen et al. (2024). Sunflower lipid content falls on the lower end of the typical range (1.5–24.6%) found in bee-pollinated crops (Vaudo et al., 2020). Schulz et al. (2000) conducted a detailed analysis of lipid compounds in sunflower pollen and detailed 35% terpenes and terpene esters, 17% diketones, 8% fatty acids (primarily 11-eicosenoic acid), 8% ketols, 12% diols, 4% dioxoalkanoic acids, 12% esters, 2% alkanes, and 2% others. Only a few studies analyzed, in detail, the profile of fatty acid content. Sunflower pollen differed in their distributions of individual fatty acids (**Table 3**). For example, stearic acid content was recorded at 1.72% (Nicolson and Human, 2013), but was also measured at 31.4% (Kostić et al., 2020). Palmitic acid had a similar variability between studies. Furthermore, α-linolenic acid, is the predominant unsaturated fatty acid (20.5–42.1%) found in sunflower pollen. In fact, sunflower pollen was reported to have a significantly greater content of linoleic and α-linolenic acid compared to almond and mixed pollen sources (Yokota et al., 2024). Sterols (i.e., β-sitosterol and 24-methylene-cholesterol) are another important group as they are not synthesized endogenously and are required to be ingested via bees’ pollen diet (Winston, 1987). Farag et al. (1980) identified the presence of cholesterol, stigmasterol, and β-sitosterol in sunflower pollen. More specifically, Takatsuto and Omote (1989) identified isofucosterol (42%), β-sitosterol (20.4%), 24-methylene-cholesterol (18.1%), 24-methylene-cholestanol (12.5%), and 23-dehydrocholesterol (4%). They identified six more phytosterols but they were all less than 1%. More recent studies on sterol identification and composition are lacking.

**Table 3:** Literature values for fatty acids reported in *Helianthus annuus* bee- and hand-collected pollen and bee bread.

*H. annuus*:	Pollen	Bee bread	References	
			Pollen	Bee bread
TFA (µg/mg)	BC: 37.1 HC: 24.2–124		BC: Nicolson and Human, 2013 ^**^ HC: Ferguson et al., 2024	
EFA (µg/mg)	HC: 26.9–51.3		HC: Ferguson et al., 2024	
SFA (% of total FA)				
Heneicosanoic	BC: 17.1		BC: Kostić et al., 2020	
Lauric	BC: 2.94/33.2 HC: 29.4/35.0	28.7	BC: Nicolson and Human, 2013 ^**^; Yang et al., 2013	Nicolson and Human, 2013
Myristic	BC: 1.17/4.60 HC: 5.27/7.93	5.20	BC: Nicolson and Human, 2013 ^**^; Yang et al., 2013	Nicolson and Human, 2013
Palmitic	BC: 12.5–43.8 HC: 21.2–23.0	26.0	BC: Kostić et al., 2020; Nicolson and Human, 2013 ^**^; Yang et al., 2013 HC: Lin and Mullin, 1999; Shakya and Bhatla, 2010	Nicolson and Human, 2013
Pentadecanoic	BC: 18.24		BC: Kostić et al., 2020	
Stearic	BC: 1.72–31.4 HC: 1.96–5.06	2.12	BC: Kostić et al., 2020; Nicolson and Human, 2013 ^**^ HC: Lin and Mullin, 1999; Shakya and Bhatla, 2010	Nicolson and Human, 2013
UFA (% of total FAs)				
α-Linolenic	BC: 20.5–42.1 HC: 16.9–22.9	19.9	BC: Kostić et al., 2020; Nicolson and Human, 2013 ^**^; Yang et al., 2013 HC: Lin and Mullin, 1999; Shakya and Bhatla, 2010	Nicolson and Human, 2013
Eicosenoic	BC: 7.25 HC: 5.78–10.5	7.38	BC: Nicolson and Human, 2013 ^**^ HC: Shakya and Bhatla, 2010	Nicolson and Human, 2013
Lignoceric	HC: 2.63		HC: Shakya and Bhatla, 2010	
Linoleic	BC: 4.45–11.9 HC: 3.57–19.7	4.80	BC: Nicolson and Human, 2013 ^**^; Yang et al., 2013 HC: Lin and Mullin, 1999; Shakya and Bhatla, 2010	Nicolson and Human, 2013
Oleic	BC: 4.10–5.87 HC: 4.06–13.3	5.91	BC: Nicolson and Human, 2013 ^**^; Yang et al., 2013 HC: Lin and Mullin, 1999; Shakya and Bhatla, 2010	Nicolson and Human, 2013

When necessary, data was extracted directly from plots using plotdigitizer.com. BC, bee-collected pollen; EFA, essential fatty acids; FA, fatty acids; HC, hand-collected pollen; SFA, saturated fatty acids; TFA, total fatty acids; UFA, unsaturated fatty acids. ^**^ Indicates the reference investigated both bee- and hand-collected pollen.

In addition to key macronutrients, pollen offers other important micronutrients, including minerals and plant secondary metabolites. Mineral nutrients are important molecular compounds needed for essential roles in physiological function, and 12 minerals (C, N, P, K, S, Ca, Mg, Na, Zn, Fe, Mn, and Cu) have been considered as biologically significant elements (Filipiak, 2019; Lau et al., 2022). In total, 17 minerals, including heavy metals, were reported present in sunflower pollen (**Table 4**). Most reported mineral nutrients had a high degree of variability. Ca, K, and P were consistently reported as major minerals in multiple studies (> 1000 mg/kg), yet they still had ranges varying 2-fold.

**Table 4:** Literature values for mineral content in *Helianthus annuus* bee- and hand-collected pollen and honey.

*H. annuus*: Minerals (mg/kg)	Pollen	Honey	References	
			Pollen	Honey
Ag		0.015		Oroian et al., 2015
Al		0.998–13.56		Ördög et al., 2017; Oroian et al., 2015; Sahinler et al., 2009
As		0.005/0.034		Ördög et al., 2017; Oroian et al., 2015
B	HC: 73.57–116.0	9.470	HC: Filipiak et al., 2022	Ördög et al., 2017
Ba		0.022/0.349		Oroian et al., 2015; Sahinler et al., 2009
Be		0.001		Oroian et al., 2015
Ca	BC: 1400–2086 HC: 2059–3264	54.28–163.9	BC: Pernal and Currie, 2000; Stanciu et al., 2011; Taha, 2015 HC: Filipiak et al., 2022	Oroian et al., 2015; Pătruică et al., 2022; Sakač et al., 2019; Tantidanai-Sungayuth et al., 2012
Cd		0.0001–0.061		Ördög et al., 2017; Oroian et al., 2015; Pătruică et al., 2022; Sahinler et al., 2009
Co	HC: 0.012–0.092	0.002–0.010	HC: Filipiak et al., 2022	Ördög et al., 2017; Oroian et al., 2015; Sahinler et al., 2009
Cr	HC: 0.036–0.177	0.003–0.108	HC: Filipiak et al., 2022	Oroian et al., 2015; Pătruică et al., 2022; Sahinler et al., 2009
Cs		0.007		Oroian et al., 2015
Cu	BC: 6.970/10.00 HC: 17.04–197.2	0.025–5.037	BC: Pernal and Currie, 2000; Taha, 2015 HC: Filipiak et al., 2022	Ördög et al., 2017; Oroian et al., 2015; Pătruică et al., 2022; Sahinler et al., 2009
Fe	BC: 27.42–343.5 HC: 24.57–211.7	0.143–24.01	BC: Pernal and Currie, 2000; Stanciu et al., 2011; Taha, 2015 HC: Filipiak et al., 2022	Ördög et al., 2017; Oroian et al., 2015; Pătruică et al., 2022; Sahinler et al., 2009; Sakač et al., 2019
Ga		0.021		Oroian et al., 2015
K	BC: 2900–6233 HC: 3901–7970	21.89–849.4	BC: Pernal and Currie, 2000; Stanciu et al., 2011; Taha, 2015 HC: Filipiak et al., 2022	Ördög et al., 2017; Oroian et al., 2015; Pătruică et al., 2022; Sahinler et al., 2009; Sakač et al., 2019; Tantidanai-Sungayuth et al., 2012
Li		13.68		Oroian et al., 2015
Mg	BC: 376.9–2705 HC: 711.6–1070	2.700–63.77	BC: Pernal and Currie, 2000; Stanciu et al., 2011; Taha, 2015 HC: Filipiak et al., 2022	Ördög et al., 2017; Oroian et al., 2015; Pătruică et al., 2022; Sahinler et al., 2009; Sakač et al., 2019; Tantidanai-Sungayuth et al., 2012
Mn	BC: 12.00/18.77 HC: 24.70–36.42	0.038–1.001	BC: Pernal and Currie, 2000; Taha, 2015 HC: Filipiak et al., 2022	Ördög et al., 2017; Oroian et al., 2015; Pătruică et al., 2022; Sahinler et al., 2009
Mo		0.010		Ördög et al., 2017
Na	BC: 46.00–6345 HC: 7.958–33.66	8.049–154.1	BC: Pernal and Currie, 2000; Taha, 2015 HC: Filipiak et al., 2022	Oroian et al., 2015; Pătruică et al., 2022; Sahinler et al., 2009; Tantidanai-Sungayuth et al., 2012
Ni	HC: 0.018–0.246	0.003–0.202	HC: Filipiak et al., 2022	Ördög et al., 2017; Oroian et al., 2015; Pătruică et al., 2022; Sahinler et al., 2009
P	BC: 2500 HC: 4952–5933	0.0001	BC: Pernal and Currie, 2000 HC: Filipiak et al., 2022	Sahinler et al., 2009
Pb		0.040–0.131		Ördög et al., 2017; Oroian et al., 2015; Pătruică et al., 2022
Rb		1.097		Oroian et al., 2015
S	BC: 1600		BC: Pernal and Currie, 2000	
Se		0.014/0.045		Ördög et al., 2017; Oroian et al., 2015
Sr	HC: 1.009–5.934	0.351	HC: Filipiak et al., 2022	Oroian et al., 2015
Tl		0.002		Oroian et al., 2015
U		0.001		Oroian et al., 2015
V	HC: 0.014–0.068	0.795	HC: Filipiak et al., 2022	Oroian et al., 2015
Zn	BC: 31.61/37.00 HC: 57.28–144.3	0.038–3.241	BC: Pernal and Currie, 2000; Stanciu et al., 2011; Taha, 2015 HC: Filipiak et al., 2022	Ördög et al., 2017; Oroian et al., 2015; Pătruică et al., 2022; Sahinler et al., 2009; Sakač et al., 2019; Tantidanai-Sungayuth et al., 2012

BC, bee-collected pollen; HC, hand-collected pollen.

The composition of plant secondary metabolites in pollen is becoming a popular topic due to their potential role in plant-pollinator interactions, bee physiology, and bee health. Sunflower pollen consists mainly of phenolamides, primarily tricoumaroyl spermidine, a diverse array of flavonoids, and phenolic acids (**Table 5**). Among phenolamides, sunflower pollen contains fewer types overall but is particularly rich in different spermidine isomers, including high levels of dicoumaroyl spermidine and tricoumaroyl spermidine (Qiao et al., 2023; Zhang et al., 2022). Flavonoids and phenolic acids are other major groups of metabolites present in sunflower pollen, particularly compounds such as apigenin, luteolin, kaempferol, quercetin glycosides, ferulic acid, and *p*-coumaric acid have been consistently reported (**Table 5**). In diverse samples of pollen from various floral resources, flavonoids accounted for more than half of the 467 total polyphenols (Rocchetti et al., 2019), and the predominant flavonoids between different floral pollen samples were mainly quercetin, isorhamnetin, and kaempferol glycosides (Qiao et al., 2023). It is important to keep in mind that the overview presented in this review does not indicate the specific isomers of each metabolite present. In fact, Qiao et al. (2023) found that the phenolics and flavonoids present were always *cis*/*trans* isomers and not in the free form. Whether this may have an impact on biological activity of these metabolites and may further influence plant-pollinator interactions is beyond the scope of this review. However, it is an interesting question that could arise when understanding the full picture of nutrition combined with how chemical variances could impact managed and wild bees’ foraging preferences.

**Table 5:** Identified plant secondary metabolites reported in the literature from *Helianthus annuus* pollen, nectar, and honey.

*H. annuus* metabolites	Pollen	Nectar	Honey
A) Phenolamides			
acylated putrescine	Palmer-Young et al., 2019	Palmer-Young et al., 2019	
dicoumaroyl putrescine	Qiao et al., 2023		
dicoumaroyl spermidine	Kyselka et al., 2018		
dicoumaroyl feruloyl spermidine	Gekière et al., 2022	Gekière et al., 2022	
tricoumaroyl spermidine	Adler et al., 2020; Gekière et al., 2022; Kyselka et al., 2018; Li et al., 2021; Lin and Mullin, 1999; Palmer-Young et al., 2019; Qiao et al., 2023	Gekière et al., 2022; Palmer-Young et al., 2019	
triferuloyl spermidine	Khongkarat et al., 2022		
tetracoumaroyl spermine	Gekière et al., 2022; Li et al., 2021; Qiao et al., 2023	Gekière et al., 2022	
tricoumaroyl feruloyl spermine	Gekière et al., 2022	Gekière et al., 2022	
B) Polyphenols			
i. Flavonoids			
a. Anthocyanins	Sharma, 2019		
b. Flavan-3-ols			
catechin			Emin Duru et al., 2023
c. Flavanones			
eriodictyol	Kostić et al., 2019		
naringenin	Kostić et al., 2019		Mattonai et al., 2016; Milosavljević et al., 2021
pinocembrin			Bobiş et al., 2021; Kečkeš et al., 2013; Milosavljević et al., 2021; Oroian and Sorina, 2017; Tomás-Barberán et al., 2001
d. Flavanonols			
taxifolin	Kostić et al., 2019		
e. Flavones			
acacetin	Kostić et al., 2019		
apigenin	Fatrcová-Šramková et al., 2016; Kostić et al., 2019		Bobiş et al., 2021; Kečkeš et al., 2013; Milosavljević et al., 2021; Oroian and Sorina, 2017
chrysin			Bobiş et al., 2021; Kečkeš et al., 2013; Milosavljević et al., 2021; Oroian and Sorina, 2017; Tomás-Barberán et al., 2001
genkwanin	Kostić et al., 2019		
luteolin	Fatrcová-Šramková et al., 2016; Kostić et al., 2019; Li et al., 2021		Bobiş et al., 2021; Kečkeš et al., 2013; Oroian and Sorina, 2017; Tomás-Barberán et al., 2001
f. Flavonols			
galangin	Kostić et al., 2019		Bobiş et al., 2021; Kečkeš et al., 2013; Milosavljević et al., 2021; Oroian and Sorina, 2017
isorhamnetin	Kostić et al., 2019		Milosavljević et al., 2021; Oroian and Sorina, 2017
isorhamnetin-3-*o*-glucoside	Kostić et al., 2019		
isorhamnetin-3-*o*-rutinoside	Kostić et al., 2019		
kaempferol	Kostić et al., 2019; Li et al., 2021; Sharma, 2019		Bobiş et al., 2021; Kečkeš et al., 2013; Milosavljević et al., 2021; Oroian and Sorina, 2017
quercetin	Fatrcová-Šramková et al., 2016; Kostić et al., 2019; Li et al., 2021; Sharma, 2019		Bobiş et al., 2021; Emin Duru et al., 2023; Kečkeš et al., 2013; Milosavljević et al., 2021; Oroian and Sorina, 2017; Tomás-Barberán et al., 2001
quercetin-3-*o*-galactoside	Kostić et al., 2019		
quercetin-3-*o*-glucoside	Lin and Mullin, 1999; Qiao et al., 2023		
quercetin-*o*-glycoside	Palmer-Young et al., 2019	Palmer-Young et al., 2019	
quercetin-3-*o*-hexoside	Adler et al., 2020		
quercetin-(*o*-malonyl)-glycoside	Palmer-Young et al., 2019	Palmer-Young et al., 2019	
quercetin-3-*o*-(6-*o*-malonyl)-hexoside	Adler et al., 2020		
quercetin-3-*o*-rhamnoside	Kostić et al., 2019		
quercetin-3-*o*-rutinoside	Kostić et al., 2019		Emin Duru et al., 2023; Kečkeš et al., 2013
myricetin	Li et al., 2021		Emin Duru et al., 2023; Oroian and Sorina, 2017
ii. Phenolic acids			
caffeic acid	Kostić et al., 2019		Bobiş et al., 2021; Dimitrova et al., 2007; Kečkeš et al., 2013; Kunat-Budzyńska et al., 2023; Milosavljević et al., 2021; Oroian and Sorina, 2017; Tomás-Barberán et al., 2001
chlorogenic acid			Bobiş et al., 2021; Emin Duru et al., 2023; Kečkeš et al., 2013; Milosavljević et al., 2021
cinnamic acid			Dimitrova et al., 2007
* o*-coumaric acid			Emin Duru et al., 2023
* p*-coumaric acid	Kostić et al., 2019; Kyselka et al., 2018		Bobiş et al., 2021; Dimitrova et al., 2007; Emin Duru et al., 2023; Kečkeš et al., 2013; Kunat-Budzyńska et al., 2023; Mattonai et al., 2016; Milosavljević et al., 2021; Oroian and Sorina, 2017; Tomás-Barberán et al., 2001
ferulic acid	Kostić et al., 2019; Kyselka et al., 2018		Dimitrova et al., 2007; Emin Duru et al., 2023; Mattonai et al., 2016; Tomás-Barberán et al., 2001
gallic acid			Emin Duru et al., 2023; Kečkeš et al., 2013; Oroian and Sorina, 2017
* p*-hydroxybenzoic acid			Bobiş et al., 2021; Dimitrova et al., 2007; Emin Duru et al., 2023; Mattonai et al., 2016; Milosavljević et al., 2021
* p*-hydroxyphenylacetic acid			Emin Duru et al., 2023
* p*-hydroxyphenylethanol			Emin Duru et al., 2023
neochlorogenic acid	Kostić et al., 2019		
phenylacetic acid			Dimitrova et al., 2007
protocatechuic acid	Kostić et al., 2019		Bobiş et al., 2021; Dimitrova et al., 2007; Emin Duru et al., 2023; Kečkeš et al., 2013; Milosavljević et al., 2021
rosmarinic acid			Emin Duru et al., 2023
syringic acid			Dimitrova et al., 2007; Kunat-Budzyńska et al., 2023
vanillic acid			Emin Duru et al., 2023; Milosavljević et al., 2021
vanillin			Emin Duru et al., 2023
iii. Others			
aesculin	Kostić et al., 2019		
coumarin			Emin Duru et al., 2023
phloretin	Kostić et al., 2019		
pinobanksin			Bobiş et al., 2021; Tomás-Barberán et al., 2001
C. Terpenes/Terpenoids			
i. Carotenoids	Fatrcová-Šramková et al., 2016		Marić et al., 2021
ii. Others			
abscisic acid			Kečkeš et al., 2013
helianyl octanoate	Schulz et al., 2000		
monoterpenol-*o*-acylglycoside		Palmer-Young et al., 2019	

This table indicates the simplified chemical name and does not specify reported isomers.

In general, both macro- and micronutrients in sunflower pollen is highly variable, making it difficult to establish a complete picture of its nutritional profile. Inconsistency exists between studies and could be due to differences in methods used for chemical analysis (e.g., Westreich and Tobin, 2021), pollen handling and storage, intraspecific variation, and/or due to an environmental × genotype interaction of the pollen producing plant. For example, pollen protein content of *Brassica* varied from 8.9–31.3% depending on if the pollen was intact or disrupted (Lau et al., 2022). Additionally, a meta-analysis by Ruedenauer et al. (2019), found that variation of pollen nutrients did not clearly correlate with phylogenetic relatedness. Zimmermann et al. (2017) found that pollen of *Poa alpina* and *P. hybrida* had higher levels of proteins and *P. hybrida* had higher carbohydrates in warmer compared to colder climates. Ultimately, with more extensive and reliable chemical analysis and synergistic protocols (e.g., Villagómez et al., 2023), future research could address if a unique physiochemical profile for sunflower pollen in different locations and cultivars could be determined. This would be immensely beneficial to predict the nutritional availability in sunflower cultivation environments for neighboring pollinator populations.

### 2.2 Nectar

Nectar chemistry studies are largely focused on sugar contents, as sugar is one of the major attractants to pollinators and is the primary energy source for foraging bees. Sunflower nectar volume and sugar mass per floret are strikingly variable across studies. Nectar volume was reported to range from 0.002–0.59 μl/floret in the staminate (male) floret stage and from 0.00–1.13 μl/floret in the pistillate (female) floret stage (**Table 6**). Sugar mass was also reported to range from 34.0–216 μg sugar/floret in the staminate stage and from 23.3–491 μg sugar/floret in the pistillate stage (**Table 6**). In terms of individual sugars, sunflower nectar is generally characterized by higher glucose (46.0–50.1%) and fructose (49.9–54.0%), and lower sucrose contents, yet some genotypes have exhibited high sucrose contents reaching up to 62% (Aquino et al., 2021; Mallinger and Prasifka, 2017b; Pham-Delègue et al., 1990, 1994; Prasifka et al., 2023; Vear et al., 1990) (**Table 6**).

**Table 6:** Literature values for volume and sugar characteristics of nectar collected from different floret stages (staminate, pistillate, or not specified) of *Helianthus annuus*.

*H. annuus*:	Staminate	Pistillate	Not specified	References		
				Staminate	Pistillate	Not specified
Nectar volume (μl/floret)	(I) 0.002–0.59	(I) 0.00–0.34 (II) 0.71–1.13	(I) 0.08–0.37; 0.10–0.78* (II) 0.02–0.16	(I) Chabert et al., 2020; Neff and Simpson, 1990; Vear et al., 1990	(I) Mallinger and Prasifka, 2017b; Neff and Simpson, 1990; Prasifka et al., 2023 (II) Hadisoesilo and Furgala, 1986	(I) Atlagić et al., 2003*; Ion et al., 2007; Joksimović et al., 2003*; Pham-Delègue et al., 1991; Zajácz et al., 2006 (II) Tepedino and Parker, 1982
Sugar mass^1^ (μg sugar/floret)	(I) 34.0–216 (III) 76.0	(I) 23.3–92.4 (II) 303–491	(I) 40.0–250 (II) 7.32–69.0 (III) 258–740	(I) Chabert et al., 2020; Vear et al., 1990 (III) Thakur et al., 2005	(I) Mallinger and Prasifka, 2017b (II) Hadisoesilo and Furgala, 1986	(I) Ion et al., 2007; Neff and Simpson, 1990; Pham-Delègue et al., 1991; Zajácz et al., 2006 (II) Tepedino and Parker, 1982 (III) Bergonzoli et al., 2022; Jocković et al., 2025
Sugar content (°Brix)	(I) 14.6–50.1	(I) 58.5–65.6 (II) 33.5–47.3	(I) 17.8–71.7 (II) 32.0–39.0	(I) Chabert et al., 2020	(I) Prasifka et al., 2023 (II) Hadisoesilo and Furgala, 1986	(I) Aquino et al., 2021; Ion et al., 2007; Pham-Delègue et al., 1991; Zajácz et al., 2006 (II) Tepedino and Parker, 1982
Sugar spectrum (%)						
Glucose	(I) 46.0–50.1		(I) 16.5–59.0 (III) 23.3–45.6	(I) Vear et al., 1990		(I) Abrol and Kapil, 1991; Aquino et al., 2021; Neff and Simpson, 1990 (III) Bergonzoli et al., 2022; Jocković et al., 2025
Fructose	(I) 49.9–54.0		(I) 9.58–46.6 (III) 43.9–53.0	(I) Vear et al., 1990		(I) Abrol and Kapil, 1991; Aquino et al., 2021; Neff and Simpson, 1990 (III) Bergonzoli et al., 2022; Jocković et al., 2025
Sucrose	(I) 0.00–1.80	(I) 0.53–62.14	(I) 4.30–66.7 (III) 1.35–2.91	(I) Vear et al., 1990	(I) Mallinger and Prasifka, 2017b; Prasifka et al., 2023	(I) Abrol and Kapil, 1991; Aquino et al., 2021; Neff and Simpson, 1990 (III) Bergonzoli et al., 2022; Jocković et al., 2025
Raffinose			(III) 2.70–6.93			(III) Bergonzoli et al., 2022
Mannitol			(III) n.d.–15.5			(III) Bergonzoli et al., 2022
Oligosaccharides (DP4 and DP5)			(III) 9.69–15.3			(III) Bergonzoli et al., 2022

Nectar collected by (I) capillary method, (II) centrifugation, and (III) washing method.

When necessary, data was extracted directly from plots using plotdigitizer.com. n.d., not detectable; DP4 and DP5: polysaccharides with 4 or 5 sugar units. ^1^To obtain comparable sugar concentration values, sugar mass was calculated based on the equation M = V·C·(0.000046·C + 0.009946) (Cruden and Hermann, 1983), where M = sugar mass in µg (or mg), V = nectar volume in nL (or µL) and C = sugar concentration in g/100 g (or °Brix). ^*^Units (mg/floret).

High variance reported for nectar volume and sugar content comes with no surprise as many studies have suggested that these characteristics are strongly influenced by abiotic factors and genotype. Studies have shown that floret stage (staminate vs. pistillate) (Chabert et al., 2020; Hadisoesilo and Furgala, 1986), vapor pressure deficit (Chabert et al., 2020), time of day (Chabert et al., 2020; Neff and Simpson, 1990; Thakur et al., 2005), temperature (Chabert et al., 2020; Prasifka et al., 2023), and fertilization treatment (Bergonzoli et al., 2022) can impact nectar volume and/or sugar concentration. Furthermore, during a long term evaluation of sunflower hybrids (2002–2010), Terzić et al. (2017) found that the growing condition caused the largest variation in nectar production. Nectar extraction, whether by centrifugation, washing method, or microcapillary method, is also suggested to influence nectar volume and sugar concentration, in which all three methods were conducted in the literature for sunflower nectar extraction (**Table 6**). For example, Mesquida et al. (1988) compared centrifugation and micropipette extraction for *Brassica napus* and found centrifugation artificially diluted the nectar samples by 4 to 6 times.

It is known that nectar contains many non-sugar metabolites, such as amino acids, lipids, vitamins, minerals, plant secondary metabolites, hormones, and proteins (Roy et al., 2017). Sunflower nectar chemistry in terms of non-sugar metabolites is lacking. It is not new knowledge that nectar contains amino acids (Baker and Baker, 1973). To the best of our knowledge, only one study identified the amino acid phenylalanine in sunflower nectar (Palmer-Young et al., 2019) and only a few studies investigated the secondary metabolite composition, in which they found various alkaloids, flavonoids, and terpenoids (Gekière et al., 2022; Palmer-Young et al., 2019) (**Table 5**). Noticeably, *Helianthus* nectar had a relatively high concentration of alkaloids compared to the other nectar sources (Palmer-Young et al., 2019). The composition of different secondary metabolites among plant structures has been found to vary. Palmer-Young et al. (2019) found that among nectar, flower, and pollen sources from 26 floral species, nectar contained the highest proportion of free amino acids and terpenoids. Nonetheless, it is important to emphasize that total amino acid content is still far higher in pollen (Nicolson, 2022) and some non-sugar metabolites in nectar might result from pollen contamination. However, Palmer-Young et al. (2019) reported that 18% of the metabolites in nectar were unique from those found only in pollen or shared between pollen and nectar. This presents an intriguing question regarding these unique nectar metabolites and whether they play a specific role in plant-pollinator interactions. This topic is almost unexplored for sunflower nectar.

Nectar is a complex topic, which is likely why we have yet to fully understand the extent of its components, mechanisms that govern its chemistry, and subsequent impacts on ecological interactions. Nectar chemistry contains more than just macro- and micronutrients, but it also contains nectar specific proteins (nectarins) in which a majority play key antimicrobial roles for nectar protection (Heil, 2011; Nepi, 2017; Roy et al., 2017). Recently, using LC-MS/MS-based comparative proteomic analysis, 144 proteins were identified in nectar of various species of *Nicotiana* (Silva et al., 2020). This study exemplifies how vast protein diversity is in nectar. They also found several proteins in nectar with roles in metabolic processes, such as regulating organic acids (malate dehydrogenase) and carbohydrate metabolism (sucrose synthase, fructokinase, and α-galactosidase). Thus, proteins in nectar impact its chemistry, which may influence its nutritional value for pollinators. Refocusing on sunflowers, unfortunately, no studies have identified or described their nectar proteins. Therefore, this would be an essential addition to the comprehensive view on the nutritional aspects of its floral resources and sunflower-pollinator interactions.

### 2.3 Bee bread

When considering honey bee nutrition, it is important to address both raw and processed floral resources. Sunflower bee bread is relatively understudied and only one study addressed its compositional parameters (**Table 1**), amino acids (**Table 2**), and fatty acids (**Table 3**) compared to fresh pollen. For example, Nicolson and Human (2013) found a significantly higher arginine concentration in bee bread, while all other macronutrient properties remained unchanged. In a separate study, Kaplan et al. (2016) analyzed sunflower bee bread and the protein content was 24.26 g/100 g, which falls on the higher end of pollen crude protein content. However, their bee bread samples contained only 45.4% *H. annuus* pollen. Whether the pollen nutritional value changes throughout the storage process is still controversial. Studies have shown no nutritional value differences (Herbert and Shimanuki, 1978; Fernandes-da-Silva and Serrão, 2000) or slightly decreased nutrition (Mayda et al., 2020). These studies did not compare fresh pollen and bee bread from the same plant source or sometimes failed to indicate the botanical origin, which is important to consider when discussing nutritional variance. Recently, Bayram et al. (2021) compared pollen and bee bread from similar plant sources and found a significantly higher concentration of protocatechuic acid, 2,5-dihydroxybenzoic acid and kaempferol in bee bread. Whereas, Degirmenci et al. (2024) and Mayda et al. (2020) found that the total phenolic content decreased. To the best of our knowledge, no studies have investigated mineral compounds and plant secondary metabolites specifically for sunflower bee bread. Similar to pollen content, it can be inferred that many factors can impact its chemical composition; for example, botanical origin, geographic location, climate, abiotic and biotic stressors, bee bread fermentation length, season, and even the presence and composition of the microbiome communities. There remains a significant gap in research comparing the compositional differences between pollen and bee bread and most research regarding such resource compositions are viewed through the human perspective. Whether these differences make raw or stored resources more nutritious for pollinators, especially bees, remains relatively unexplored.

### 2.4 Honey

Inside the honey bee hive, the conversion of nectar to honey offers many advantages as a stored energy and nutrient source. It serves to maintain colonies though the winter months and aids nurse bees with an in-hive source for their own care and for feeding the larval brood (Crane, 1975; Winston, 1987). When classifying honey, it is of course important to consider both the chemical and physical properties and potentially one of the most important factors is the nectar chemistry from where it originates (Crane, 1975). It is critical to consider that physicochemical properties of honey differ based on natural variation; thus the entire physical and chemical picture of honey are matched to the ‘ideal reference model’ of that botanical source (Persano Oddo and Bogdanov, 2004). The reference model of classifying honey has further challenges as the legislation for honey criteria and standards depend on the country (Thrasyvoulou et al., 2018). Sunflower honey has generally been described as having high glucose content (Cotte et al., 2004a; Juan-Borrás et al., 2014; Persano Oddo et al., 1995; Persano Oddo and Piro, 2004), proline, and acidity values (Persano Oddo and Piro, 2004). Pollen content of sunflower honey was also found with extreme variability from 20–90% (Persano Oddo and Piro, 2004). However, this could be explained by country regulation as Germany requires > 50% pollen content, whereas Greece requires > 20% (Thrasyvoulou et al., 2018).

In general, based on our compilation of sunflower honey studies, the general sugar content ranged from 73.1–80.8% and sixteen different sugars have been identified (**Table 7**). Threonine (Cotte et al., 2004b) and phenylalanine (Kečkeš et al., 2013) were also reported higher in sunflower honey compared to other honey types (**Table 2**), and K and Mg were higher compared to rapeseed and black locust honeys (Ördög et al., 2017) (**Table 4**). In contrast to pollen, nectar, and bee bread, many studies have investigated the specific secondary metabolites found in sunflower honey. The metabolite profile is diverse, primarily consisting of polyphenols, specifically flavonoids and phenolic acids (**Table 5**). The compounds identified varied between studies and sunflower honey samples. Mattonai et al. (2016) consistently found *p*-hydroxybenzoic acid, *p*-coumaric acid, ferulic acid, and naringenin in all sunflower honey samples. The overall concentration of flavonoids and phenolics (**Table 8**) had large ranges from 19.5–269 mg QE/100 g and 6.90–854 mg GAE/100 g, respectively. This also depended on the study as Pătruică et al. (2022) found higher concentration of phenolic acids in Romanian sunflower honey compared to other literature sources. As noted, honey is characterized by its’ antioxidant and antimicrobial properties, with the latter discussed in greater detail in a subsequent section. Interpreting honey’s antioxidant properties is challenging due to the lack of standardized methods and the use of various methodologies with differing units (**Table 9**). Castiglioni et al. (2017) observed that antioxidant levels varied depending on the free radical scavenging assay, such that some samples showing significant differences in ABTS but not in DPPH. This highlights the need for standardized procedures, as antioxidant values can only be relatively compared under consistent testing conditions.

**Table 7:** Literature values for sugar characteristics of *Helianthus annuus* honey.

*H. annuus* honey	%	References
Total sugar content	73.1–80.8	Bath and Singh, 1999; Erler et al., 2014; Gherman et al., 2014; Oroian et al., 2017; Oroian and Sorina, 2017
Glucose	31.2–45.0 379^*^	Bobiş et al., 2021; Cotte et al., 2004a; Devillers et al., 2004; Erler et al., 2014; Gherman et al., 2014; Juan-Borrás et al., 2014; Kádár et al., 2010; Kunat-Budzyńska et al., 2023; Marić et al., 2021; Mateo and Bosch-Reig, 1997, 1998; Oroian et al., 2015, 2017; Persano Oddo et al., 1995; Persano Oddo and Piro, 2004; Sakač et al., 2019; Zhelyazkova and Lazarov, 2017
Fructose	34.6–45.3 400^*^	Bobiş et al., 2021; Cotte et al., 2004a; Devillers et al., 2004; Erler et al., 2014; Gherman et al., 2014; Juan-Borrás et al., 2014; Kádár et al., 2010; Kunat-Budzyńska et al., 2023; Marić et al., 2021; Mateo and Bosch-Reig, 1997, 1998; Oroian et al., 2015, 2017; Persano Oddo et al., 1995; Persano Oddo and Piro, 2004; Sakač et al., 2019; Zhelyazkova and Lazarov, 2017
Sucrose	n.d.–6.46	Bobiş et al., 2021; Cotte et al., 2004a; Devillers et al., 2004; Erler et al., 2014; Gherman et al., 2014; Isopescu et al., 2014; Juan-Borrás et al., 2014; Kádár et al., 2010; Kunat-Budzyńska et al., 2023; Manolova et al., 2021; Mateo and Bosch-Reig, 1997, 1998; Milosavljević et al., 2021; Persano Oddo et al., 1995; Sahinler et al., 2009; Sari and Ayyildiz, 2012; Zhelyazkova and Lazarov, 2017
Erlose	0.20–0.69	Devillers et al., 2004; Gherman et al., 2014; Kunat-Budzyńska et al., 2023
Fucose	0.35	Kunat-Budzyńska et al., 2023
Isomaltose	n.d.–0.31	Cotte et al., 2004a; Erler et al., 2014; Gherman et al., 2014; Mateo and Bosch-Reig, 1997, 1998; Persano Oddo et al., 1995
Kojibiose	1.55/1.58	Mateo and Bosch-Reig, 1997, 1998
Laminaribiose	0.50	Cotte et al., 2004a
Maltose	0.90–2.74	Cotte et al., 2004a; Erler et al., 2014; Gherman et al., 2014; Mateo and Bosch-Reig, 1997, 1998; Persano Oddo et al., 1995
Maltotriose	0.10	Cotte et al., 2004a
Maltulose	0.50–0.77	Cotte et al., 2004a; Mateo and Bosch-Reig, 1997, 1998
Melezitose	n.d.	Devillers et al., 2004
Neo-kestose	0.10	Cotte et al., 2004a
Palatinose	0.10	Cotte et al., 2004a
Panose	0.10	Cotte et al., 2004a
Raffinose	n.d.	Devillers et al., 2004
Rhamnose	1.55	Kunat-Budzyńska et al., 2023
Trehalose	0.35/0.60	Cotte et al., 2004a; Gherman et al., 2014
Turanose	0.90–1.10	Cotte et al., 2004a; Erler et al., 2014; Gherman et al., 2014

n.d., not detectable. ^*^Units (g/kg).

**Table 8:** Literature values of compositional parameters reported for *Helianthus annuus* honey.

*H. annuus* honey	Values	References
Acidity (free) (meq/kg)	13.0–47.3	Bath and Singh, 1999; Devillers et al., 2004; Erler et al., 2014; Lazarević et al., 2012; Marić et al., 2021; Milosavljević et al., 2021; Oroian and Sorina, 2017; Oroian et al., 2017; Pauliuc and Oroian, 2020; Persano Oddo and Piro, 2004; Sakač et al., 2019; Truzzi et al., 2014; Vîjan et al., 2023
Acidity (total) (meq/kg)	23.9–70.0	Bath and Singh, 1999; Erler et al., 2014; Manolova et al., 2021; Persano Oddo et al., 1995; Sahinler et al., 2009; Sari and Ayyildiz, 2012; Truzzi et al., 2014
Ash (%)	0.10–0.36	Milosavljević et al., 2021; Oroian and Sorina, 2017; Oroian et al., 2017; Pătruică et al., 2022; Persano Oddo et al., 1995; Sahinler et al., 2009; Sakač et al., 2019; Vîjan et al., 2023
Electrical conductivity (mS/cm)	0.19–0.78	Bobiş et al., 2021; Devillers et al., 2004; Juan-Borrás et al., 2014; Kádár et al., 2010; Kunat-Budzyńska et al., 2023; Lazarević et al., 2012; Manolova et al., 2021; Marić et al., 2021; Mateo and Bosch-Reig, 1998; Milosavljević et al., 2021; Oroian and Sorina, 2017; Oroian et al., 2015, 2017; Pauliuc and Oroian, 2020; Persano Oddo et al., 1995; Persano Oddo and Piro, 2004; Sahinler et al., 2009; Sakač et al., 2019; Truzzi et al., 2014; Vîjan et al., 2023; Zhelyazkova and Lazarov, 2017
HMF (mg/kg)	1.00–25.5	Bobiş et al., 2021; Devillers et al., 2004; Erler et al., 2014; Isopescu et al., 2014; Juan-Borrás et al., 2014; Kádár et al., 2010; Manolova et al., 2021; Marić et al., 2021; Milosavljević et al., 2021; Oroian et al., 2015; Pauliuc and Oroian, 2020; Persano Oddo et al., 1995; Sahinler et al., 2009; Sakač et al., 2019; Truzzi et al., 2014; Vîjan et al., 2023; Zhelyazkova and Lazarov, 2017
Moisture content (%)	14.7–20.4	Bath and Singh, 1999; Bobiş et al., 2021; Devillers et al., 2004; Erler et al., 2014; Isopescu et al., 2014; Kunat-Budzyńska et al., 2023; Lazarević et al., 2012; Manolova et al., 2021; Marić et al., 2021; Mateo and Bosch-Reig, 1998; Milosavljević et al., 2021; Oroian and Sorina, 2017; Oroian et al., 2015, 2017; Pătruică et al., 2022; Pauliuc and Oroian, 2020; Persano Oddo et al., 1995; Sahinler et al., 2009; Sakač et al., 2019; Sari and Ayyildiz, 2012; Truzzi et al., 2014; Vîjan et al., 2023; Zhelyazkova and Lazarov, 2017
Total flavonoid content (mg QE/100 g)	19.5–269 10.9–63.2^*^	Bobiş et al., 2021; Erler et al., 2014; Marić et al., 2021 ^*^; Pătruică et al., 2022; Predescu et al., 2015 ^*^; Vîjan et al., 2023 ^*^
Total phenolic content (mg GAE/100 g)	6.90–854	Atanacković Krstonošić et al., 2019; Bobiş et al., 2021; Castiglioni et al., 2017; Erler et al., 2014; Kunat-Budzyńska et al., 2023; Marić et al., 2021; Milosavljević et al., 2021; Pătruică et al., 2022; Predescu et al., 2015; Sari and Ayyildiz, 2012; Vîjan et al., 2023

CE, catechin equivalents; GAE, gallic acid equivalents; QE, quercetin equivalents.^*^Units (mg CE/100 g).

**Table 9:** Literature values of antioxidant capacity measured with various methods reported for *Helianthus annuus* honey.

*H. annuus* honey	Values	References
i. DPPH (IC_50_) (g/ml)	0.018–0.322	Atanacković Krstonošić et al., 2019; Emin Duru et al., 2023; Marić et al., 2021
ii. DPPH-Vis method (mmol Trolox equivalents/kg)	0.49	Castiglioni et al., 2017
iii. DPPH-EPR method (mmol Trolox equivalents/kg)	0.44	Castiglioni et al., 2017
iv. DPPH (%)	60.2–78.3	Pătruică et al., 2022; Pauliuc and Oroian, 2020; Predescu et al., 2015
v. DPPH (% inhibition of 2,2-diphenyl-1-picrylhydrazyl)	23.1–65.4	Bobiş et al., 2021; Erler et al., 2014; Sari and Ayyildiz, 2012; Vîjan et al., 2023
vi. ABTS assay (mmol Trolox equivalents/kg)	4.70	Castiglioni et al., 2017

As mentioned, summarizing sunflower honey’s characteristics is challenging due to the many physical and chemical properties considered in honey classification, inconsistencies reported across studies, and differing country legislations. Furthermore, honey characteristics are impacted by the botanical origin, year and their interaction (Vîjan et al., 2023); intra-sample variation (Mattonai et al., 2016), and climate (Kečkeš et al., 2013). It is also known that sugars in honey can be influenced by factors such as the ripening period and storage conditions (Crane, 1975). Many studies lack clarity regarding their processing methods, ripening duration, and storage conditions before measuring sugar characteristics; which further complicates the overall picture.

## 3 Sunflower as a nutritional resource for bee development and health

### 3.1 Pollen

**Figure 2:**
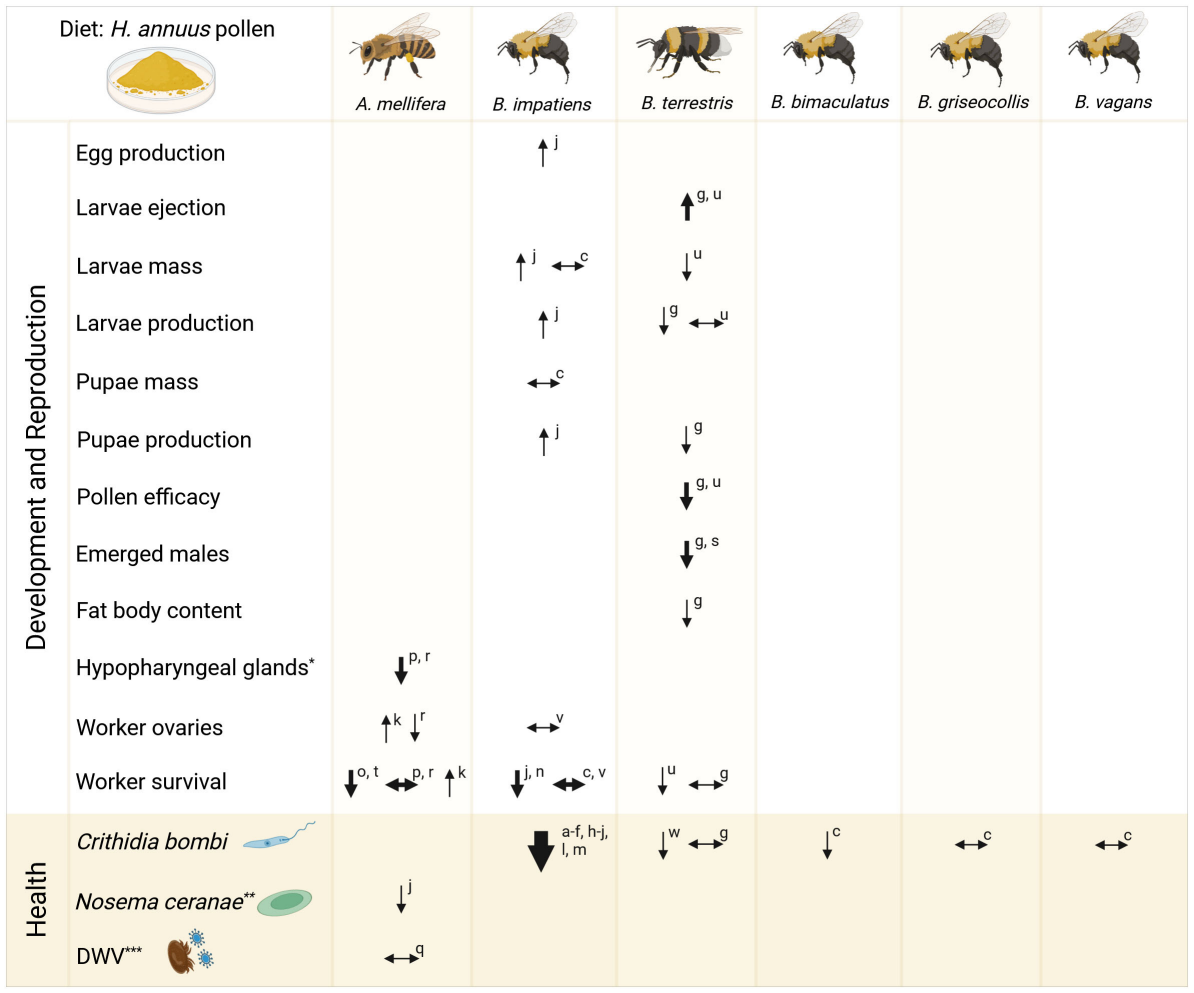
Sunflower pollen diet on bee development and health. Reported literature results on the impact of *Helianthus annuus* pollen diet on survival, development, physiological, and health parameters in experimental queen-less cage assays. Arrow thickness indicates the number of studies for the corresponding parameter (thin: 1 study; medium: 2 studies; thick: > 10 studies). ^*^Measured as protein content or volume; ^**^recently renamed *Vairimorpha ceranae*; ^***^DWV, Deformed wing virus transmitted by *Varroa destructor*. Created in BioRender Reference letters: **(a)** Adler et al., 2020; **(b)** Figueroa et al., 2023; **(c)** Fowler et al., 2022a; **(d)** Fowler et al., 2023; **(e)** Fowler et al., 2022b; **(f)** Fowler et al., 2020; **(g)** Gekière et al., 2022; **(h)** Giacomini et al., 2023; **(i)** Giacomini et al., 2021; **(j)** Giacomini et al., 2018; **(k)** Human et al., 2007; **(l)** LoCascio et al., 2019a; **(m)** LoCascio et al., 2019b; **(n)** McAulay and Forrest, 2019; **(o)** Nicolson et al., 2018; **(p)** Omar et al., 2017; **(q)** Palmer-Young et al., 2023; **(r)** Pernal and Currie, 2000; **(s)** Regali and Rasmont, 1995; **(t)** Schmidt et al., 1995; **(u)** Tasei and Aupinel, 2008; **(v)** Treanore et al., 2019; **(w)** Vanderplanck et al., 2023.

The increasing cultivation of sunflowers across Europe highlights the need to understand their nutritional impact on pollinator communities and identify areas for further research. Sunflower pollen is often reported as a low quality nutritional resource for honey bees, as a result of lower levels of protein (Nicolson and Human, 2013; Pamminger et al., 2019a) and a few essential amino acids reported below minimum requirements for honey bees (Nicolson and Human, 2013; Taha et al., 2019). One commonly used method for testing the nutritional quality of pollen is done by using controlled feeding cage assays and are primarily focused on the western honey bee, *Apis mellifera;* the common eastern bumble bee, *Bombus impatiens*; and the buff-tailed bumble bee, *B. terrestris*, which is not surprising due to their economic importance, commercial rearing and availability. **Figure 2** summarizes the results from literature investigating sunflower pollen diets on bee reproductive, development, and health parameters, including antiparasitic, antimicrobial, and antiviral effects. Cage experiments with a pure sunflower pollen diet often resulted in negative reproductive parameters for *B. terrestris*, including reduced mass and production of larvae and pupae; and reduced male emergence (Gekière et al., 2022; Regali and Rasmont, 1995; Tasei and Aupinel, 2008). In queen-right *B. terrestris* colonies, Zhou et al. (2024) found negative developmental effects in those fed sunflower and buckwheat pollen, including decreased worker mass and number, reduced wing length, and absence of a reproductive phase. On the contrary, one study investigated *B. impatiens* and found an increase in egg, larvae, and pupae production (Giacomini et al., 2018). Negative impacts on development were also observed by reduced fat body content in *B. terrestris* (Gekière et al., 2022) and reduced hypopharyngeal gland protein content or volume in *A. mellifera* (Omar et al., 2017; Pernal and Currie, 2000). However, varying results were found for worker ovary development within *A. mellifera* and *B. impatiens*. Even more interesting was the extensive inconsistency of worker survival within and between the investigated bee species.

Overall the variable results, partial investigations for *A. mellifera* and *B. impatiens*, and lack of research regarding wild bee species, the nutritional impact of sunflower pollen is still not fully understood. In general, there is limited research on the potential impact of specific nutritional components on reproduction, development, cognition, and even foraging preferences. It was found that a deficiency in omega-3 fatty acid resulted in smaller hypopharyngeal glands (Arien et al., 2015), omega-3 and oleic acid deficiencies reduced learning abilities (Arien et al., 2015; Muth et al., 2018), and high ratios of omega-6:3 was detrimental to cognitive performance (Arien et al., 2018). Pollen sterol content was also found to be a key nutrient for bumble bee larvae growth, likely due to their role in hormone synthesis and cell membrane function (Moerman et al., 2017). Regarding pollen mineral nutrients, Filipiak and Filipiak (2020) found that K deficiency reduced survival and impaired cocoon development for the mason bee *Osmia bicornis*.

Assessing colony health encompasses more than just reproductive and developmental metrics, but also evaluating their resistance and susceptibility to pathogens and parasites. Numerous studies have challenged the perception of sunflower pollen as “low-quality”, as it consistently shows a positive health impact by reducing the gut parasite *Crithidia bombi* in *Bombus* species, particularly *B. impatiens* (**Figure 2**). Sunflower pollen’s impact on *C. bombi* has been discovered repetitively for *B. impatiens*; nevertheless, variable results were found investigating sunflower pollen on *C. bombi* infection intensity in *B. terrestris* (Gekière et al., 2022; Vanderplanck et al., 2023) and only one study found variable results between various wild *Bombus* species (Fowler et al., 2022a). Not much is known regarding the impact on other pathogens or parasites. *Nosema ceranae* (recently renamed *Vairimorpha ceranae)* cell counts decreased in *A. mellifera* fed sunflower pollen, but it increased mortality as if they underwent pollen starvation (Giacomini et al., 2018). Furthermore, one study reported no impact on infection intensity of deformed wing virus (DWV) in sunflower fed honey bees (Palmer-Young et al., 2023).

**Figure 3:**
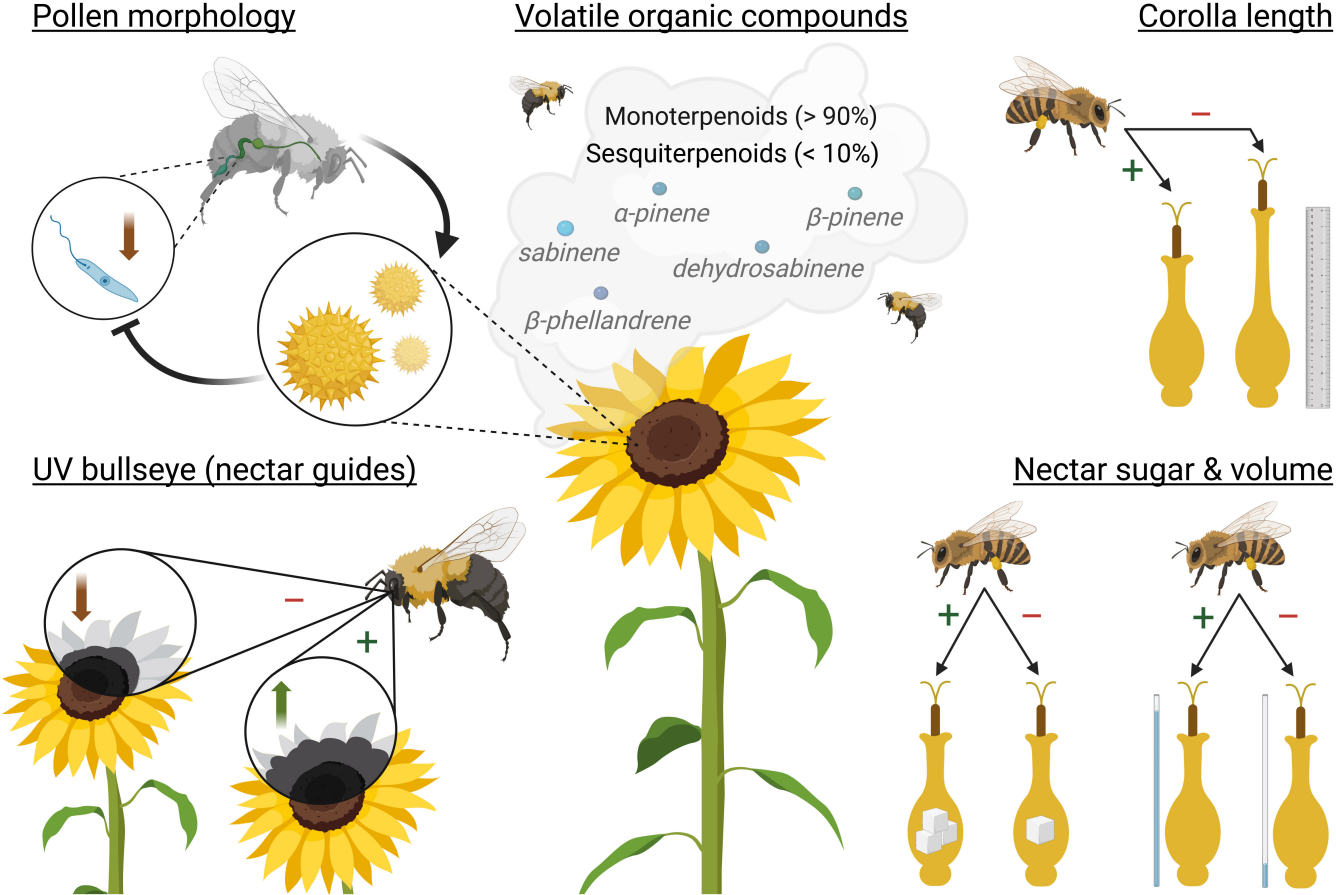
Key sunflower pollination syndromes. Clockwise from the top left with respective section in review: sunflower pollen consumption on *Crithidia bombi* infection intensity in *Bombus* spp. (section 3.1); dominant sunflower volatile organic compounds (section 4.2); corolla length on pollinator attraction (section 4.1); sunflower nectar sugar content and volume on pollinator attraction (section 4.4); and increased UV bullseye size (nectar guides) on pollinator attraction (section 4.1). Plus indicates increased pollinator attraction and minus indicates less attraction. Created in BioRender.

Many studies have tested whether the effect from sunflower pollen was due to specific pollen nutrient components, such as individual fatty acids or secondary metabolites rutin or tricoumaroyl spermidine (Adler et al., 2020) or other unique phenolamides (Gekière et al., 2022). Furthermore, Yost et al. (2023) had the hypothesis that bumble bees fed sunflower pollen might have an influenced gut microbiome impacting pathogen resistance. They fed *B. impatiens* (inoculated with *C. bombi*) a solution of guts dissected from bumble bees fed either sunflower or buckwheat pollen while on a control diet of wildflower pollen. However, they found no differences in *C. bombi* cell counts between the recipient bees fed either gut solution. Interestingly, Giacomini et al. (2023) found that sunflower pollen consumption upregulated immune transcripts linked to the maintenance and repair of gut epithelial cells, theorizing pollen’s role in disrupting infection from *C. bombi* by potentially removing or preventing the attachment of flagellated pathogens. Figueroa et al.’s (2023) clever study design found that sunflower exines alone reduced infection intensity (along with 4 of 7 additional Asteraceae species compared to non-Asteraceae pollen); thus, supporting the role of spiny morphology for infection suppression (**Figure 3**). Yet, a reduction in *C. bombi* infection intensity was not present in all spiny pollen treatments (Figueroa et al., 2023). If the morphological features play a large role in infection intensity, further research needs to be done regarding more pollen external features, for example: spine density, spine length, and pollen grain size.

With the focus on the morphological aspect of sunflower pollen, Knoerr et al. (2024) followed up this finding on whether the spiny structure could result in intestinal damage. They found that sunflower exines added to diets resulted in no differences in *B. impatiens* mortality or intestinal damage, however those fed spines reduced larval and microcolony weights. As suggested by Knoerr et al. (2024), these negative findings could be a consequence of larvae consuming higher amounts of empty exines, rather than full pollen grains. Likewise, Vanderplanck et al. (2020) found no intestinal damage in the digestive tract of *B. terrestris* fed spiny whole *Taraxacum* pollen, but they also reported higher larval ejection compared to non-spiny treatments. Ultimately, the studies addressing *C. bombi* are limited mostly to *B. impatiens* (**Figure 2**); therefore, a knowledge gap persists for species specific interactions. This is particularly relevant given that both sunflower and *B. impatiens* are native to North America. Thus far, developmental parameters appear to be less detrimental, and the medicinal benefits of sunflower pollen more consistent for *B. impatiens* compared to *B. terrestris*. Further research is needed to explore these interactions in the context of sunflowers impact on native vs. non-native pollinators.

Major limitations of the studies summarized in **Figure 2** include their confinement to laboratory environments, the use of queen-less bumble bee colonies, variations in pollen collection methods used for feeding (hand- vs. honey bee-collected pollen), and comparisons between sunflower pollen and other pollen sources that differ in seasonal availability (often constrained by commercial availability). This further complicates efforts to obtain a clear picture on the impacts of sunflower pollen consumption. Only a few studies have investigated field realistic conditions. Charrière et al. (2010) found no difference in brood production between their honey bee colonies placed next to sunflower fields and control colonies placed 3 km away. Queen production of *B. impatiens* increased 30% with every order of magnitude increase in sunflower area (Malfi et al., 2023). Considering sunflowers apparent medicinal properties, an increase in sunflower area was also associated with reduced *C. bombi* prevalence, however no effect on *Apicystis bombi* or the black queen cell virus (BQCV) was found (Malfi et al., 2023). It was also found that every 10-fold increase in sunflower area decreased *C. bombi* infection intensity 23.2% (Giacomini et al., 2018) and a two-fold increase in sunflower area resulted in a 28% decrease in *Varroa* mite infestation for *A. mellifera*, without effects on *Nosema ceranae* (Palmer-Young et al., 2023). These studies are difficult to associate these effects solely to sunflower resource availability. Either these studies did not consider how much sunflower pollen was collected or they found relatively low quantities. Malfi et al. (2023) recorded that only 8.5% of bumble bees carried sunflower pollen compared to 46% carrying other Asteraceae pollen, but this was proportional to the *Helianthus annuus* cover compared to other flowering sources. Charrière et al. (2010) found that their treatment colonies placed at the edge of sunflower fields collected only 2.2–3% sunflower pollen in 2003, and 11–38% in 2004, depending on the colony and location, as they favored other sources despite sunflower abundance. This highlights a key point that should not be overlooked, such that lab experiments only provide a snapshot on how colony health may be impacted. They do not show colonies ability to compensate for nutritional imbalances or the realistic conditions that pollinators forage on a diversity of pollen sources. As previously mentioned, it is known that mono-diets are not considered well-balanced diets for social bees (Alaux et al., 2010; Di Pasquale et al., 2013, 2016; Leponiemi et al., 2023), presumably also not for solitary bees. Given that bees forage from a variety of pollen sources, it’s unclear if a single source, like sunflower pollen, can be classified as health detrimental or beneficial.

### 3.2 Nectar

When assessing the nutritional impact of floral resources on bees, it is crucial to consider more than just pollen. Bees’ diets also include nectar, and some species consume bee bread and honey, all of which contribute to their overall nutrition. Except the basic nutritional summary, no studies have investigated sunflower nectar consumption on pollinator nutrition. Nectar nutritional studies often focus on the predominant secondary metabolites found in nectar from various plant species, using commercially sourced metabolites for experimentation. The consumption of anabasine, catalpol, nicotine, and thymol reduced the cell count of *C. bombi* infection in *B. impatiens* (Richardson et al., 2015); as well as a reduced fecal intensity of *C. bombi* from an alkaloid, gelsemine, found in nectar (Manson et al., 2010). Moreover, abscisic acid enhanced immune responses in *Apis mellifera* (Negri et al., 2015). On that note, it cannot be ignored that metabolites in sunflower nectar could also contribute to bee physiology and health, though we first need to understand the composition of non-sugar metabolites offered.

### 3.3 Bee bread and honey

Stored pollen and nectar in the hive provide essential support to workers, offering resources during times of need and aiding honey bee workers which are conducting their roles inside the hive. Of course, the question arises on whether these stored resources might offer an advantage in their nutritional nature compared to raw nectar and pollen. As previously mentioned, bee bread is debated on whether it differs in nutritional quality compared to pollen. Only one study investigated sunflower pollen vs. bee bread and found no impacts on *A. mellifera*s’ survival or extraction efficiency (digestion efficiency of individual pollen grains), meaning that pollen was not digested better in either fresh or stored form (Nicolson et al., 2018). Despite some studies supporting the notion that pollen and bee bread do differ (see section 2.3), there is no current support indicating this difference has realistic impacts for bee fitness.

Similarly, this knowledge gap holds true for sunflower honey. Honey offers unique properties in terms of its antimicrobial nature. The antimicrobial properties come from its’ high sugar content which results in an osmotic pressure dehydrating bacterial cells (Molan, 1992); an accumulation of hydrogen peroxide from the glucose oxidase system (White et al., 1963); and the presence of phenolic acids and flavonoids (Čižmárik and Matel, 1970; Ferreres et al., 1994). An expansive nutritional profile for sunflower honey exists, presumably since honey is consumed by humans. Our knowledge on how honey’s high antimicrobial and antioxidant properties can enhance honey bee health is limited. Studies have shown that sunflower honey displays mild antimicrobial activity against *Staphylococcus aureus* (Emin Duru et al., 2023), *Escherichia coli, Candida parapsilopsis, C. albicans, Listeria monocitogenes*, and *Bacillus cereus* (Pătruică et al., 2022). Thus, it is hypothesized that honey could have medicinal properties against bee relevant bacteria or pathogens. Gherman et al. (2014) observed in a choice assay using honey bees, that honey bees infected with *Nosema ceranae* increasing preferred sunflower honey with increasing infection intensity compared to honeydew honey. Furthermore, they also showed that sunflower honey fed honey bees had significantly lower *N. ceranae* spore load compared to honeydew honey. In a bacterial growth inhibitory study, sunflower honey inhibited American foulbrood bacterial strains more effectively than black locust honey and completely inhibited *Paenibacillus alvei* growth; yet, black locust honey was more effective on reducing European foulbrood related bacteria (Erler et al., 2014). Based on this study, the antimicrobial and antibiotic properties of honey seems to be honey and bacterial strain specific. It could be worth exploring how the availability of various floral resources and subsequent honey types present in a honey bee hive might drive the pathogen susceptibility of a colony, or whether they can selectively consume honey of a certain nature depending on need. Understanding this interaction could provide useful colony health indicators for scientists and beekeepers.

## 4 Drivers of sunflower-pollinator interaction

### 4.1 Floral morphology and pigments

This review has primarily focused on sunflower pollination syndromes that provide nutrition to pollinators, primarily bees. However, pollinator preferences and foraging decisions are influenced by the association of the reward, potentially the nutritional quality, and to certain floral traits (i.e., flower size, color, scent, etc.) (Chittka and Raine, 2006; Frachon et al., 2021; Knauer and Schiestl, 2015). Sunflower ligules, the modified petals on the outermost part of the inflorescence, often appear yellow or orange due to the presence of secondary metabolites, primarily carotenoids (responsible for yellow to orange pigmentation) and flavonoids. Flavonoids are a diverse group of phenolic secondary metabolites encompassing anthocyanins (orange, blue, purple, or even black hues), and other groups, such as flavonols and flavones, which provide pale yellow hues or are mostly colorless to the human eye (reviewed by Iwashina, 2015). The regulation of ligule color in sunflower has been recently proposed to be governed by the HaMYBA-HabHLH1 and HaMYBF complex which regulates anthocyanin and flavonol accumulation, respectively (Jiang et al., 2024). Likewise, Ma et al. (2024) reported HanMYB1 plays a role in regulation of anthocyanin accumulation. They found that chrysanthemin and epigallocatechin are major anthocyanins in red sunflowers and rutin and kaempferol are major flavones and flavonols in yellow sunflowers. See Galiseo et al.’s (2024) review on the identified carotenoid compounds found in *H. annuus*.

Flower pigments that are most interesting to pollinator attraction are the ultraviolet (UV)-absorbing pigments that are not visible to humans, but are responsible for UV patterns on flowers called nectar guides (Leonard and Papaj, 2011; Penny, 1983; Todesco et al., 2022). UV-absorbing pigments accumulate in the ligules base and the outermost edge reflects UV radiation resulting in a bullseye pattern visible to foraging bees (Moyers et al. 2017; Todesco et al. 2022). These UV-absorbing pigments consist primarily from flavonols and flavones. In particular for sunflower, biosynthesis of flavonol glycoside pigments is governed by the transcription factor, HaMYB111 (Todesco et al., 2022). In an elaborate study to test bullseye pattern size and pollinator attraction, Todesco et al. (2022) found that within 1484 individuals from 106 *H. annuus* populations, individuals with intermediate UV patterns had the highest pollinator visitation rates compared to small or large patterns (**Figure 3**). Similarly, *Rudbeckia* flowers with smaller bullseye patterns (reduced nectar guides) had significantly less pollinator visitation (Horth et al., 2014).

Accumulation of these flavonoids have been shown to vary tremendously in the *Helianthus* genus and between *H. annuus* individuals (Scogin, 1978; Todesco et al., 2022). Thus, questions arise regarding their ecological significance. Todesco et al. (2022) found that the bullseye patterns are correlated to geoclimate factors, which is not surprising as flavonols are involved in mitigating abiotic stressors (reviewed in Shomali et al., 2022). Larger UV bullseyes were present in colder environments; however the variability in size was predominantly correlated to lower humidity climates. This suggests flavonoids’ dual role for pollinator attraction and water conservation in sunflowers.

Floral color and nectar guides facilitate visual identification, but upon floral visitation there are additional features that can enable easier access to the floral reward. The sunflower inflorescence consists of many individual flowers, disc florets, each formed by a corolla composed of 5 fused petals. Inside the corolla are the male and female reproductive parts and the nectary is at the base (Sammataro et al., 1985). Many studies have found that the corolla length of sunflower has an impact on pollinator visitation frequency (**Figure 3**). Shorter corollas, thus easier nectar access, increased both wild bee and honey bee visits (du Toit and Coetzer, 1991; Ferguson et al., 2024; Mallinger and Prasifka, 2017b; Portlas et al., 2018). Ferguson et al. (2021) also found that bee community differed based on corolla length relative to tongue length. Most bees preferred shorter corollas, whereas *Bombus* spp. showed a preference for sunflower lines with larger florets. Significant foraging preferences relative to corolla length was also reported dependent on the year or sowing period (Ferguson et al., 2021, 2024).

Ultimately, sunflower-pollinator interactions have been shown to be impacted by floral UV patterns and corolla length, but research is lacking in regards to other floral traits. For example, sunflowers are known to have a high density of glandular trichomes present on both leaves and florets, and have been shown to produce allelochemicals which may be important for plant defense again pathogens and herbivores (Brentan Silva et al., 2017). Sesquiterpene lactones are present in glandular trichomes of sunflower and have been shown to defend against insect pests (Chou and Mullin, 1993; Mullin et al., 1991; Prasifka et al., 2015). Little research has been done on trichomes impact on pollinators. It has been theorized that oleiferous trichomes in *Bulbophyllum saltatorium* might play a role in attracting pollinators (Stpiczyńska et al., 2018). This is an important future topic to address through the pollinator perspective, as we cannot exclude the influence of sunflower trichomes on their interactions.

### 4.2 Olfactory attraction

Pollinators use both visual and olfactory cues to navigate the complicated environment (Burger et al., 2010; Leonard et al., 2012). Volatile organic compounds (VOCs) include a vast diversity of terpenes, among those are monoterpenoids and sesquiterpenoids which play a major role in floral scent (Pichersky and Raguso, 2018). We are only beginning to unravel their complexity and interactions with the biotic environment, including their roles in attracting or repelling pollinators and herbivores (Raguso, 2008; Pichersky and Raguso, 2018; Slavković and Bendahmane, 2023; Wright and Schiestl, 2009). Research regarding sunflower VOCs began in the late 1980s to early 1990s with a few studies identifying potential pollinator discriminatory compounds (Pham-Delègue et al., 1986, 1990; Thiery et al., 1990). A long gap succeeded this research and only recently interest has surged, likely driven by growing sunflower breeding efforts, leading to extensive identification of between 100 to 500 VOCs in wild and cultivated *Helianthus* species (Anandappa et al., 2023; Bahmani et al., 2022, 2023; Jocković et al., 2024). Few studies have also addressed sunflower VOCs to understand plant-pest interactions for thrips (Qu et al., 2024) and yellow peach moth (Zhou et al., 2023).

Based on recent studies, the volatile profile of petals or disc florets of wild and domesticated *H. annuus* were dominated by monoterpenoids (> 90%), followed by sesquiterpenoids (< 10%), and even fewer non-terpene compounds (Anandappa et al., 2023; Bahmani et al., 2022, 2023). Of the monoterpenoids, α-pinene always had the highest relative abundance (Anandappa et al., 2023; Bahmani et al., 2022, 2023; Qu et al., 2024; Zhou et al., 2023). This consistency is surprising considering studies have investigated different floral tissues (petals vs. disc florets), used different sample preparation (fresh vs. frozen), and analysis methods. Thus, monoterpenoid-sesquiterpenoid balance and the high abundance of α-pinene appears characteristic for *H. annuus* (**Figure 3**). Though there is some variance in the less abundant VOCs. Cultivated *H. annuus* contained 6–20% sabinene, which was only present in 1% or less of wild *H. annuus* (Anandappa et al., 2023). They also found higher proportions of VOCs in cultivated *H. annuus* which are considered pleasant to human smell, such as D-limonene, eucalyptol, and β­phellandrene (Anandappa et al., 2023). The second most prevalent monoterpenoid was either sabinene (Anandappa et al., 2023; Bahmani et al., 2023), dehydrosabinene (Bahmani et al., 2022), β-pinene (Qu et al., 2024), or β-­phellandrene (Zhou et al., 2023) (**Figure 3**). The remaining major VOCs reported across *H. annuus* studies were D-limonene, β-pinene, and γ-terpinene (Bahmani et al., 2022); D-limonene, (+)-calarene, and germacrene D (Qu et al., 2024); β-pinene, D-limonene, α-terpinene, γ-terpinene, terpinene-4-ol, o-cymene, and bornyl acetate (Bahmani et al., 2023); and camphene, β-pinene, 3-carene, D-limonene, eucalyptol, γ-terpinene, and valencene (Zhou et al., 2023).

The total relative abundance of VOCs between wild and cultivated *H. annuus* is up for debate. Bahmani et al. (2022, 2023) found that total VOC abundance was significantly lower in cultivated sunflowers, and sesquiterpenoids were reduced by 6–8 times. However, this was not supported by Anandappa et al. (2023). The potential impact of reduced volatile abundance on pollinator attraction and decreased sesquiterpenoids on herbivore defense in cultivated sunflowers warrants further investigation (see Prasifka et al., 2015). Overall, *H. annuus* appears to have relatively consistent compound diversity and monoterpenoid-sesquiterpenoid balance. This is not the case when looking at other species in the *Helianthus* genus. Species are found to vary in their floral monoterpenoid-sesquiterpenoid composition, compound abundance, and major volatiles (Bahmani et al., 2022; Jocković et al., 2024). Bahmani et al. (2022) also found that among the wild *Helianthus* species, higher monoterpenoids were found in arid climates for annuals and erect perennials, and sesquiterpenoids were higher in mesic habitats for erect perennials. Interestingly, Todesco et al. (2022) found that arid climates had larger nectar guides to attract pollinators, proposing this was driven by geoclimate conditions. Nonetheless, it is an interesting connection, as arid climates may have both increased monoterpenoids and larger nectar guides.

Pollen and nectar VOCs are less studied but are important, likely contributing to the floral VOC profile and both serve as a key nutritional resources for pollinators. No studies have addressed nectar specific VOCs, and only a few have for pollen. Bertoli et al. (2011) found a greater abundance of sesquiterpenoids compared to monoterpenoids. The major VOCs consisted of the sesquiterpenoids β-elemene, β-gurjunene, β-chamigrene, germacrene D, and *trans*-γ-cadinene, as well as the monoterpenoids α-pinene oxide, *trans*-verbenol, pinocarvone, myrtenol, verbenone, and isobornyl acetate. In contrast, Qu et al. (2024) found that the major compounds mirrored their VOCs reported in disc florets, with the exception of α-terpinene and γ-selinene found in pollen. Leaf VOCs are another dimension to consider for plant-insect interactions. As our focus is on floral structures, we exclude them here; see Galisteo et al. (2024) for a review on sunflower terpenoid diversity.

Identifying VOCs driving plant-pollinator interactions is challenging, as production and emission are complex, and VOCs often exist in a bouquet rather than single compounds. We are just starting to understand which specific compound or mix of VOCs correspond to pollinator attraction or deterrence. Studies from Pham-Delègue et al. (1986, 1990) and Thiery et al. (1990) identified a fraction of compounds isolated from VOCs of sunflower that either elicited behavior or antennae responses by honey bees. When comparing their proposed “pollinator recognizable” compounds to recently reported major floral and pollen VOCs in sunflower, only bornyl acetate, eucalyptol, and germacrene D were found in floral VOCs, while verbenone, β-elemene, myrtenol, and germacrene D were found in pollen. In other studies, Blight et al. (1997) found eight VOCs from *Brassica napus* flowers were recognized by honey bees. Those that also occurred as major VOCs in sunflower were α-pinene, α-terpinene, and 3-carene. Farina et al. (2020) used the mechanism of associative bee learning with floral rewards to elicit a recruitment behavior of honey bees towards sunflower fields by feeding honey bee colonies with sugar syrup scented with sabinene, beta-pinene and limonene (see also Estravis-Barcala et al., 2021; Farina et al., 2023), all three identified as major sunflower VOCs. Nonetheless, the highest abundance does not necessarily indicate the most relevant, as volatiles with low abundance or their combinations may also be important, as either attractive or deterrents. In particular, honey bees are able to detect subtle differences in the ratio of two odors, and identify individual compounds in complex floral scents (Wright and Schiestl, 2009). Although not related to sunflowers, recent studies have identified some VOCs as either innate attractants or repellents to bees. For example, ß-trans-bergamotene was found to be an innate “dishonest” attractant for bumble bees, as they preferred it in higher amounts even from non-rewarding flowers (Haber et al., 2019). Conversely, repellent VOCs discovered were α- and β-selinenes found in carrot genotypes (Quarrell et al., 2023); E-2-hexanal, Z-3-hexenol, and Z-3-hexenyl acetate found in strawberry cultivars (Ceuppens et al., 2015; Klatt et al., 2013); and dioxolanes, piperidines and organosulfur compounds found in onion genotypes (Soto et al., 2015). The evidence we have for VOCs and pollinators is quite limited to laboratory settings which do not directly translate to real environment interactions. There is a lot more we can learn from understanding the chemical world, including those pertaining to sunflower-pollinator interactions.

### 4.3 Pollen

We do not know much about what drives specific choices of pollen collection by pollinators, let alone understanding how the nutritional components of particular species of pollen might influence foraging decisions. For sunflowers, studies have made more observational links to foraging preferences based on sunflower pollen production. Such that, honey bees have shown to exhibit preference to male sterile sunflowers (lacking pollen) (Estravis Barcala et al., 2019; Mallinger and Prasifka, 2017b; Pham-Delègue et al., 1990; Skinner, 1987; Tepedino and Parker, 1982), whereas *Melissodes* spp. and other wild bees were most abundant on male fertile sunflowers (Estravis Barcala et al., 2019; Mallinger and Prasifka, 2017b; Tepedino and Parker, 1982). Furthermore, few studies have addressed foraging preferences linked to pollen chemistry. Ferguson et al. (2024) investigated the fatty acid content of pollen across different sunflower lines to examine its association with pollinator visitation. However, they found high variability between years, suggesting that the nutritional composition of sunflower pollen may be more variable than previously expected. Although not specific to sunflower, foraging preferences were linked to protein:lipid (P:L) ratios, which differed among bee species. *B. impatiens* favored higher P:L ratios in the range from 4:1 to 10:1 (Vaudo et al., 2016, 2020), while honey bees preferred 1:1 and 2:1 ratios (Vaudo et al., 2020). The nutritional requirements of pollinators may influence their foraging preferences. However, linking specific nutritional compounds to these choices is highly complex, as various plant attraction mechanisms, such as nectar, flower color, morphology, and olfactory compounds, also play a role.

It is known that pollen is a more specialized resource to collect than nectar. Pollen’s primary function is for plant reproduction; thus there is inherent conflict to the double function of pollen, between plant reproduction and food resources for the Anthophila clade (Michez et al., 2012; Murray et al., 2018). It is therefore logical that pollen contains defenses to deter herbivores and over consumers (pollen defense on bee ecology reviewed in Rivest and Forrest, 2020). Major pollen defenses are hypothesized to be chemical; however, very few studies have linked plant secondary metabolites, mostly alkaloids, to negative pollinator effects (see Rivest and Forrest, 2020; Rivest et al., 2024). Furthermore, Asteraceae species like sunflower, have spined pollen which may act as a morphological defense against consumption and may influence the visiting pollinator community (see Rivest et al., 2024), but this topic needs more research. How the bioactive properties or morphological aspects of pollen can govern foraging preferences is not well understood (see Tourbez et al., 2023; Vanderplanck et al., 2019). Especially in regards to how these factors may drive foraging preferences based on nutritional needs, pollinator specific adaptions, or potential medicinal benefits (as discussed in section 3.1).

### 4.4 Nectar

Focusing on the interactions between nectar traits and pollinators, nectar volume and sugar content are key drivers influencing visitation in sunflowers (**Figure 3**). Wild bees and honey bees increased visitation with increased total sugar content (Estravis Barcala et al., 2019; Mallinger and Prasifka, 2017b), but not by the specific sugar composition of nectar (Mallinger and Prasifka, 2017b, see also Soto et al., 2021 with onion). In contrast, Pham-Delègue et al. (1990), assumed sucrose to be the main driver of visitation in honey bees, but this was concluded based on testing only two parental lines of two hybrids. Pham-Delègue et al. (1990) did not measure additional floral characteristics or nectar volume, thus visitations cannot be only associated to sucrose content. Nectar volume and total sugar content are influenced by genotype, environment, and their interactions (Chabert et al., 2020; Terzić et al., 2017). Many studies have found significant differences between foraging preferences among different sunflower genotypes (Bergonzoli et al., 2022; Cerrutti and Pontet, 2016; Chambó et al., 2011; Fell, 1986; Sapir, 2009; Stejskalová et al., 2018). With numerous studies indicating variations in cultivar attractiveness, genetic mapping of traits responsible for nectar production and chemistry is emerging as a key focus in sunflower breeding efforts aimed at enhancing plant appeal to pollinators. Two homologous genes, ARF8 and DAD1, governing nectar production in *Arabidopsis*, have been reported as candidate genes governing sunflower nectar volume (Barstow et al., 2022). Additionally, HaCWINV2 gene, putatively involved in sucrose metabolism, had a strong linear association between gene expression and glucose and fructose content (Aquino et al., 2021). Despite the significant influence of genetic × environmental interactions, sunflower breeding efforts have identified strong genotype effects (Chabert et al., 2020; Terzić et al., 2017). Consequently, the breeding of sunflowers with both stable and specific nectar volume and sugar quantity and quality remains appealing for optimizing floral resources for bees.

Nectar is no longer seen as a simple sugar reward for pollinators, but in fact has a complicated chemistry. Recent literature focused on the presence of amino acids, nectarins, and both attractive and toxic secondary metabolites (reviewed in the following studies, Adler, 2000; Barberis et al., 2023; Heil, 2011; Nepi, 2017; Roy et al., 2017). In general, though not specifically for sunflowers, numerous nectar studies investigated the diverse roles of specific chemical compounds in herbivore defense, antimicrobial activity, health benefits, and their physiological effects on pollinator cognition and foraging preferences (Barberis et al., 2023). Honey bees showed a preference for phenylalanine enriched solutions (Hendriksma et al., 2014) and varying concentrations of proline (Carter et al., 2006). Further intriguing findings revealed the presence of components of the honey bee queen mandibular pheromone in the nectar of buckwheat and Mexican sunflower, *Tithonia diversifolia* (Liu et al., 2015). They found that in an artificial nectar solution, *Apis cerana* exhibited a preference for these compounds. Nicotine and caffeine consumption in nectar have been shown to enhance foraging memory and learning (Baracchi et al., 2017; Wright et al., 2013). Specifically for caffeine, its concentration plays a crucial role. Wright et al. (2013) demonstrated that caffeine enhances bees’ memory for floral rewards, whereas at higher concentrations, such as those found in *Citrus × meyeri*, it elicited an aversive response in bees (Muth et al., 2022). The complexity continues as Muth et al. (2022) further showed that two insect neurotransmitters regulating foraging behaviors found in nectar (octopamine and tyramine), when present with caffeine, eliminated bees distaste to caffeine rich floral nectar.

Specific deterrent properties of nectar have been associated to presence of potassium in some onion and avocado genotypes (Afik et al., 2006a, b; Hernández et al., 2019; Soto et al., 2013) and the terpenoid triptolide, which reduced olfactory learning and memory (Zhang et al., 2018). These deterrents have the dual effect of (*i*) reducing the palatability of nectar and (*ii*) altering the associated learning of floral cues with the presence of such compounds. As has been shown with caffeine, we know very little about nectar modulators and whether deterrent properties in nectar are counteracted by other compounds influencing pollinator visitation.

Nectar chemistry is also complicated to investigate due to other interesting and understudied factors, such as the presence and community of floral microbes. To the best of our knowledge no literature has investigated the nectar microbiome of sunflower nectar. Various microbe nectar specialists including yeasts (i.e., *Metschnikowia* spp.) and bacteria (i.e., *Acinetobacter* spp. and *Rosenbergiella* spp.) have been identified and may play roles on altering floral attraction and foraging preferences, or impacting the nutritional availability of nectar to pollinators (reviewed in Martin et al., 2022; Quevedo-Caraballo et al., 2025; Vannette, 2020). In particular, yeasts, *Merremia aegyptia* and *Cordia sebestena*, significantly affected the concentration of individual nectar sugars and their proportions (Canto and Herrera, 2012). *Metschnikowia reukaufii* was found to be a deterrent to honey bees and decreased the concentration of amino acids and increased the amount of volatiles emitted in nectar (Rering et al., 2021). Nectar bacteria, *Acinetobacter*, can stimulate pollen germination and bursting (Christensen et al., 2021), potentially influencing the nutritional availability to pollinators. How the precise interactions guiding synergistic or antagonistic relationships between plant and microbe and further extended to pollinators needs more study.

## 5 Conclusion

Sunflower pollen and nectar show significant variability in their macro- and micronutrient chemistry. Nectar chemistry is a complicated blend of various compounds, but our knowledge of sunflower nectar is limited to sugar compounds with much still left to be studied. Additionally, inconsistency was reported for developmental parameters measured after bee consumption of sunflower pollen. Both aspects of variability in nutrition and subsequent bee development makes it challenging to determine the nutritional potential sunflowers may provide neighboring pollinator communities. Despite these variable and undefined nutritional aspects, it has been continuously reported that sunflower pollen has a medicinal effect on reducing *C. bombi* in *Bombus* spp. This encourages an additional factor to consider when defining the quality of sunflower floral resources. The extent of sunflower-pollinator interaction goes beyond nutrition, as we know that certain sunflower cultivars seem to have increased pollinator attractiveness, presumably from key pollination syndromes (**Figure 3**). Ultimately, with the rise of breeding efforts and sunflower cultivation as a bee-friendly crop, it is important to continue understanding the balance between sunflower as a nutritional source versus its’ attractive pollination syndromes.
